# Real-world pharmacovigilance insights into drug-induced risk of alopecia

**DOI:** 10.3389/fphar.2025.1703423

**Published:** 2025-11-28

**Authors:** Huixiang Li, Haijian Wei, Qiaoqiao Shentu, Jianxin Cui, Jiaojiao Chen

**Affiliations:** 1 Department of Pharmacy, Yantai Yuhuangding Hospital Affiliated to Qingdao University, Yantai, Shandong, China; 2 Department of Organ Transplantation, Yantai Yuhuangding Hospital Affiliated to Qingdao University, Yantai, Shandong, China; 3 Department of pharmacy, Dongyang Red Cross Hospital, Dongyang, Zhejiang, China

**Keywords:** drug-induced alopecia, pharmacovigilance, FAERS, disproportionality analysis, time-to-onset, risk assessment

## Abstract

**Background:**

Alopecia is a significant adverse effect that profoundly impacts quality of life. Although numerous medications are implicated, the real-world risk profiles across drug classes and patient demographics remain poorly quantified.

**Objective:**

To identify and characterize drugs associated with alopecia using real-world data from the FDA Adverse Event Reporting System (FAERS).

**Methods:**

FAERS reports from Q1 2004 to Q4 2024 were analyzed using four disproportionality methods (ROR, PRR, BCPNN, MGPS) to detect signals of drug-alopecia associations. Subgroup analyses were conducted by age, gender, and drug category. Time-to-onset (TTO) was analyzed using the Weibull distribution model.

**Results:**

A total of 181,838 reports with drug-associated alopecia were identified. The mean age was 53.84 ± 16.28 years, and 76.82% of reports were from females. Oncology medications showed strongest association (37.5%), especially docetaxel (ROR = 70.38). Endocrine (18.8%) and immune system medications (10.9%) were also prominent. The TTO analysis revealed a bimodal distribution, with 40.2% of cases occurring within 30 days and 13.1% manifesting at 240–360 days. Males experienced a significantly shorter onset latency compared to females (108 days vs. 236 days, *P* < 0.001). Oncology drugs also showed shorter latency than non-oncology agents (198 vs. 308 days, *P* < 0.001). Notably, comparison with United States prescribing information revealed that 23.4% of high-signal drugs lacked documentation of alopecia in their official labels.

**Conclusion:**

This large-scale pharmacovigilance study identified 64 drugs with significant alopecia signals, highlighting distinct demographic patterns and latency periods. The findings underscore the need for heightened clinical vigilance, gender-specific monitoring, and updates to labels to better reflect real-world risks.

## 1 Introduction

Alopecia, characterized by abnormal hair loss, significantly impairs quality of life (Vary, 2015; Kearney et al., 2025). It encompasses various clinical types, among which androgenetic alopecia (AGA) and alopecia areata (AA) are the most prevalent (Alkhalifah et al., 2010; Mubki et al., 2014). AGA exhibits an extremely high prevalence, affecting approximately 80% of men and 50% of women by age 70 (Devjani et al., 2023; Gupta et al., 2025). As the second most common type, the incidence rate of AA has increased significantly in recent years, particularly among children and adolescents (McKenzie et al., 2022). Despite not being life-threatening, alopecia profoundly impacts psychological wellbeing and social functioning (Hunt and McHale, 2005; Muntyanu et al., 2023).

The etiology of alopecia is complex and multifactorial, involving genetic, hormonal, autoimmune, nutritional, and environmental factors (Pratt et al., 2017; Kinoshita-Ise et al., 2023). Critically, medications are recognized as important modifiable triggers. (Alhanshali et al., 2023; Ezemma et al., 2024; Ravipati et al., 2024). Drug-induced alopecia (DIA) typically presents as non-scarring, diffuse hair loss. It primarily occurs through two mechanisms: anagen effluvium, involving rapid hair loss due to direct cytotoxicity (e.g., chemotherapy), and the more common telogen effluvium, characterized by delayed, gradual shedding. (Piraccini et al., 2006; Trüeb, 2010). A key clinical challenge is identifying the causative drug, often complicated by variable latency periods and concurrent use of multiple medications.

The FDA Adverse Event Reporting System (FAERS), which collates adverse events (AE) data from healthcare professionals, patients, and manufacturers, provides a valuable resource for detecting drug safety signals in real-world populations (Sakaeda et al., 2013). While Hill et al. previously described drug-induced hair loss using FAERS data, their study did not include quantitative analyses stratified by key variables like age, gender, or drug category, nor did it identify specific risk signals for DIA (Hill et al., 2025). Therefore, our study aimed to systematically identify and characterize DIA signals through comprehensive disproportionality analyses to delineate risk profiles and provide actionable clinical insights.

## 2 Methods

### 2.1 Data source and processing

The data for this study were extracted from FAERS database, covering reports from the first quarter of 2004 (Q1 2004) to the fourth quarter of 2024 (Q4 2024). Raw quarterly ASCII files were downloaded from the FAERS public repository (https://fis.fda.gov/extensions/FPD-QDE-FAERS/FPD-QDE-FAERS.html). The downloaded data were imported into SAS software (version 9.4) for cleaning and statistical analysis. To ensure data integrity, a structured de-duplication process was applied. Duplicate reports were identified and removed as recommended by the FDA (Tregunno et al., 2014). Specifically, for reports sharing the same CASEID, the record with the most recent FDA_DT was retained. If the CASEID and FDA_DT were the same, the report with the highest PRIMARYID was retained.

### 2.2 Identification of target AE reports

In the FAERS database, AE reports are coded using the preferred terms (PT) from the Medical Dictionary for Regulatory Activities (MedDRA). To identify reports of alopecia, we employed Standardized MedDRA Queries (SMQs) (MedDRA v26.0) in this study. SMQs are predefined sets of PTs that represent related clinical conditions or syndromes (Mozzicato, 2007), with two search modalities: broad-scope search and narrow-scope search. In this study, we adopted the narrow-scope search, restricting inclusion to PTs with a well-established relationship to alopecia (Supplementary Table S1). AE reports were considered as target cases if they contained any PTs specified in Supplementary Table S1. Crucially, to address potential confounding from polypharmacy (multiple drugs reported per case) and to focus on the associations with the highest suspicion, our analysis was strategically restricted to drugs designated as the “Primary Suspect” in the reported events. Reports listing the drug of interest as “Secondary Suspect”, “Concomitant”, or “Interacting” were excluded. Furthermore, each case report contributed only once to the disproportionality analysis for a specific drug, and no statistical weighting was applied for the presence of other drugs within the same report. This conservative approach prioritizes signal specificity over sensitivity, aiming to highlight the most robust drug-event associations.

### 2.3 Detection of ADR signals

Disproportionality analysis is a special data mining algorithm developed for the quantitative detection of ADR signals in large pharmacovigilance databases (Yan et al., 2022). Based on the standard 2 × 2 contingency table (Supplementary Table S2), this method quantifies the disparity between the observed frequency and the expected background frequency for a specific drug-AE pair, thereby establishing potential statistical associations between drugs and AEs (Li et al., 2024). In this study, we employed four disproportionality analysis methods to detect positive signals of drug-associated alopecia: reporting odds ratio (ROR), proportional reporting ratio (PRR), Bayesian confidence propagation neural network (BCPNN), and multi-item gamma Poisson shrinker (MGPS) (Candore et al., 2015). Positive signal criteria are provided in Supplementary Table S3. To strengthen evidence for robust association between the identified drugs and alopecia, a drug was selected as a positive signal only if it met the criteria of all four methods simultaneously. Subgroup analyses stratified by gender and age were conducted to explore the potential variation in drug-associated alopecia signals across these demographic dimensions. P-values from these subgroup analyses were adjusted for multiple comparisons using the Bonferroni correction, with statistical significance defined by the corrected threshold. Furthermore, in order to conduct a more in-depth investigation into the relationship between the positive drugs and AEs, stratification analyses were performed based on number of reports, drug category, and signal intensity. Additionally, to characterize the latency period of drug-associated alopecia, the Weibull distribution model was used to analyze the time-to-onset (TTO) of AEs for each drug (Kinoshita et al., 2020). We further compared differences in TTO distributions across drug categories and between genders. Finally, to assess the alignment between our pharmacovigilance signals and established safety labels, we further verified the signals by checking for adverse reactions in the official United States prescribing information (FDA’s Drugs@FDA search database). For each drug, we systematically examined the “Adverse Reactions” section of the current, approved label for any mention of “alopecia”, “hair loss”, or related terms.

### 2.4 Statistical analysis

Descriptive analysis was performed to summarize and present the clinical characteristics of the patients in drug-associated alopecia reports. Categorical variables were presented as frequencies and percentages, while continuous variables were expressed as median with interquartile range (IQR) or mean ± standard deviation (SD) based on distribution normality. Between-group comparisons of the TTO distributions were performed across drug categories and gender strata using non-parametric tests (Wilcoxon rank-sum test). Statistical analysis was conducted using a combination of software tools, including Microsoft Excel 2019, Origin (version 2021), SPSS (version 23.0; IBM, United States), GraphPad Prism (version 8.0.2) and R (version 4.2.3), where *p* < 0.05 was considered statistically significant.

## 3 Results

### 3.1 Descriptive analysis of drug-associated alopecia reports

**FIGURE 1:**
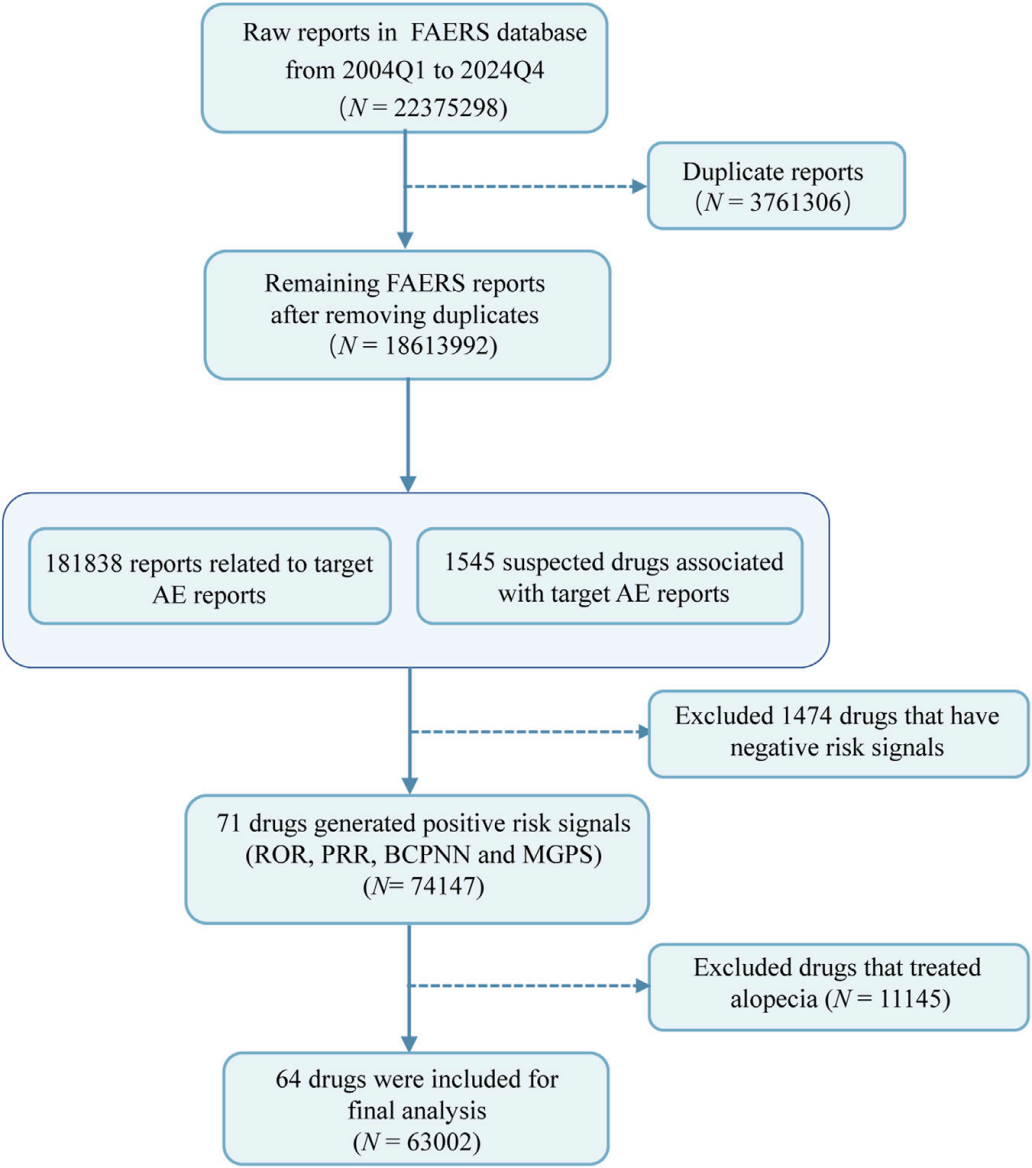
Flowchart for cleaning process for drug-associated alopecia data in the FAERS database. Abbreviations: FAERS, Food and Drug Administration Adverse Event Reporting; ROR, reporting odds ratio; PRR, proportional reporting ratio; BCPNN, Bayesian confidence propagation neural network; MGPS, Multi-item Gamma Poisson Shrinker.

**FIGURE 2:**
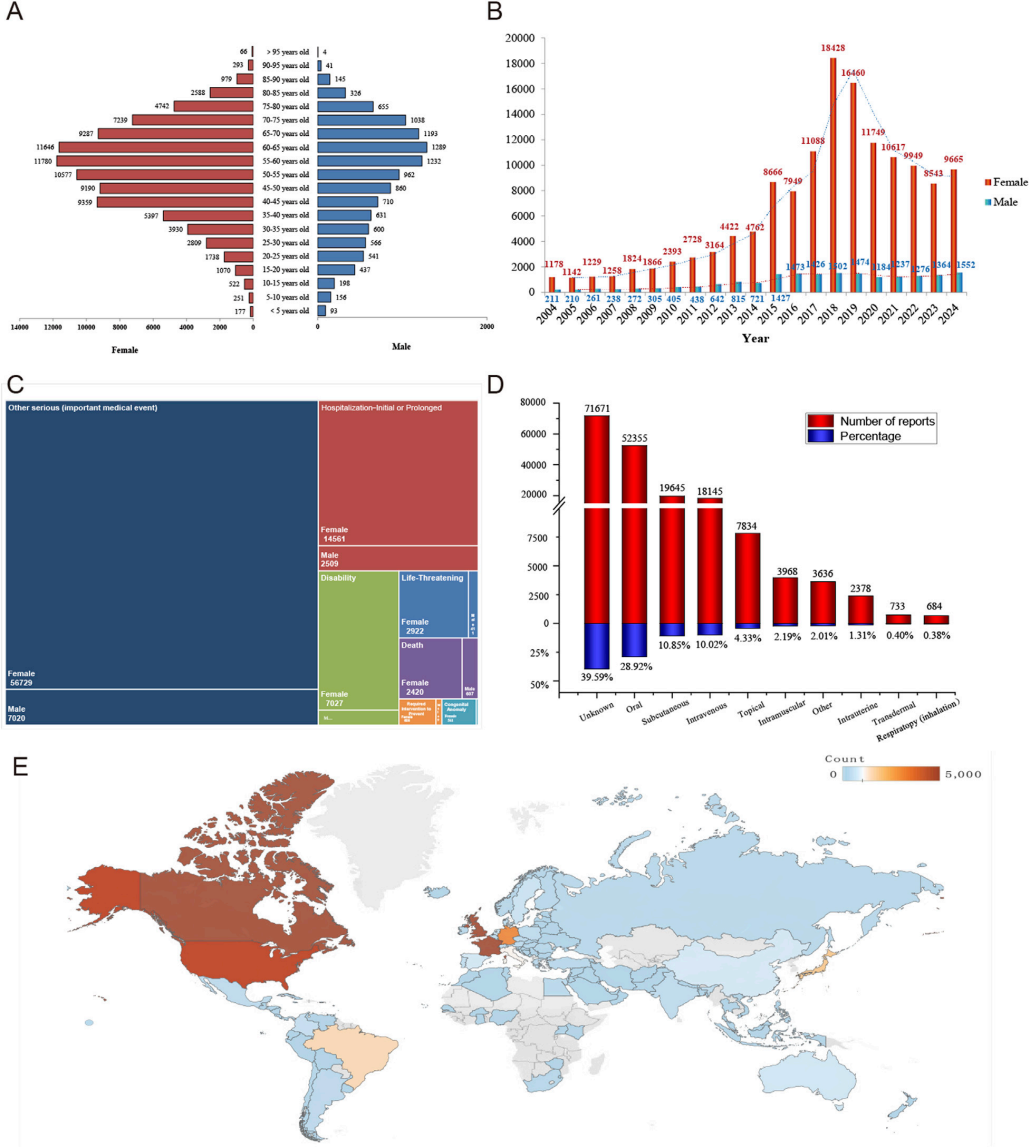
Baseline characteristics of adverse reactions associated with alopecia. **(A)** Population pyramid of patients with drug-associated alopecia categorized by gender and age. **(B)** Bar chart showing the annual reporting counts of drug-associated alopecia by gender. **(C)** Distribution of adverse reaction outcomes for patients with drug-associated alopecia categorized by gender. **(D)** Bar chart of drug administration routes for patients with drug-associated alopecia. **(E)** Heatmap of reporting countries for patients with drug-associated alopecia.

From Q1 2004 to Q4 2024, the FAERS database recorded a total of 22,375,298 patients reporting AEs, of which 181,049 patients were reported to have experienced drug-associated alopecia, involving 181,838 reports. Figure 1 illustrated the detailed data processing. And Table 1 presented the clinical characteristics of AEs associated with alopecia. The mean age of the patients was 53.84 ± 16.28 years, with females accounting for the majority at 76.82%. Specifically, in females, the reported age for drug-associated alopecia was primarily concentrated in the 50–65 years range, whereas in males, it was concentrated in the 55–70 years range (Figure 2A). Furthermore, we observed a gradual increase in the number of reported cases of drug-related alopecia over the years, peaking in 2018 and subsequently showing a downward trend in the past 6 years. Notably, the incidence was significantly higher in females than in males annually (Figure 2B). The patients had a median weight of 72.00 kg (IQR 60.33–86.18 kg). Consumers were the main reporters, accounting for 55.88%. In terms of the severity of the AE reports, the proportions of patients with non-serious and serious reports were roughly similar, at 53.31% and 46.69% respectively. As for the outcomes of the aforementioned AEs, the most frequent outcome was “Other Serious (important medical event)” (66.21%: 56,729 females, 7,020 males), followed by “Hospitalization-Initial or Prolonged” (16.84%: 14,561 females, 2,509 males) (Figure 2C). Among patients with known administration routes, oral (28.92%), subcutaneous (10.85%), and intravenous (10.02%) administration were the predominant routes (Figure 2D). Regarding reporting region, the United States submitted the majority of reports, followed by Canada and United Kingdom, with 134,199, 13,238 and 5,380 reports, respectively (Figure 2E). For more details, please refer to Figure 2 and Table 1.

**TABLE 1:** Baseline data of drug-related alopecia patients reported in FAERS database.

Characteristics		Values
Gender		
Female	*n* (%)	139,084 (76.82)
Male	*n* (%)	18,433 (10.18)
Unknown	*n* (%)	23,532 (13.00)
Age (year)		
All of known	Mean ± SD Median (IQR)	53.84 ± 16.28 55.00 (43.00, 65.00)
Female	Mean ± SD Median (IQR)	54.18 ± 15.73 55.00 (44.00, 65.00)
Male	Mean ± SD Median (IQR)	51.34 ± 19.61 55.00 (37.00, 66.00)
Unknown	*n* (%)	74,984 (41.42)
Weight (Kg)		
All of known	Mean ± SD Median (IQR)	76.42 ± 280.33 72.00 (60.33, 86.18)
Female	Mean ± SD Median (IQR)	75.92 ± 296.93 70.30 (59.80, 85.81)
Male	Mean ± SD Median (IQR)	80.64 ± 24.63 79.00 (68.40, 90.72)
Unknown	*n* (%)	129,508 (71.53)
Reporting year		
2004Q1-2010Q4	*n* (%)	13,601 (7.51)
2011Q1-2015Q4	*n* (%)	29,375 (16.22)
2016Q1-2020Q4	*n* (%)	87,379 (48.26)
2021Q1-2024Q4	*n* (%)	50,694 (28.00)
Reporting region		
United States	*n* (%)	134,199 (74.12)
Canada	*n* (%)	13,238 (7.31)
United Kingdom	*n* (%)	5,380 (2.97)
France	*n* (%)	5,136 (2.84)
Germany	*n* (%)	3,168 (1.75)
Occupation of reporters		
Consumer	*n* (%)	101,167 (55.88)
Physician	*n* (%)	24,722 (13.65)
Pharmacist	*n* (%)	21,587 (11.92)
Lawyer	*n* (%)	15,753 (8.70)
Other health-professional	*n* (%)	12,092 (6.68)
Unknown	*n* (%)	5,728 (3.16)
Severity		
Non-serious	*n* (%)	96,511 (53.31)
Serious	*n* (%)	84,538 (46.69)
Outcome		
Life-threatening	*n* (%)	3,769 (2.08)
Hospitalization-initial or prolonged	*n* (%)	18,354 (10.14)
Disability	*n* (%)	9,746 (5.38)
Death	*n* (%)	3,544 (1.96)
Congenital anomaly	*n* (%)	692 (0.38)
Required intervention to prevent permanent impairment/Damage	*n* (%)	721 (0.40)
Other serious (important medical event)	*n* (%)	72,151 (39.85)

Continuous numerical variables are expressed as mean ± standard deviation median (Q1, Q3). Categorical variables are presented as n (%).

### 3.2 Distribution of drug categories associated with alopecia signals

**FIGURE 3:**
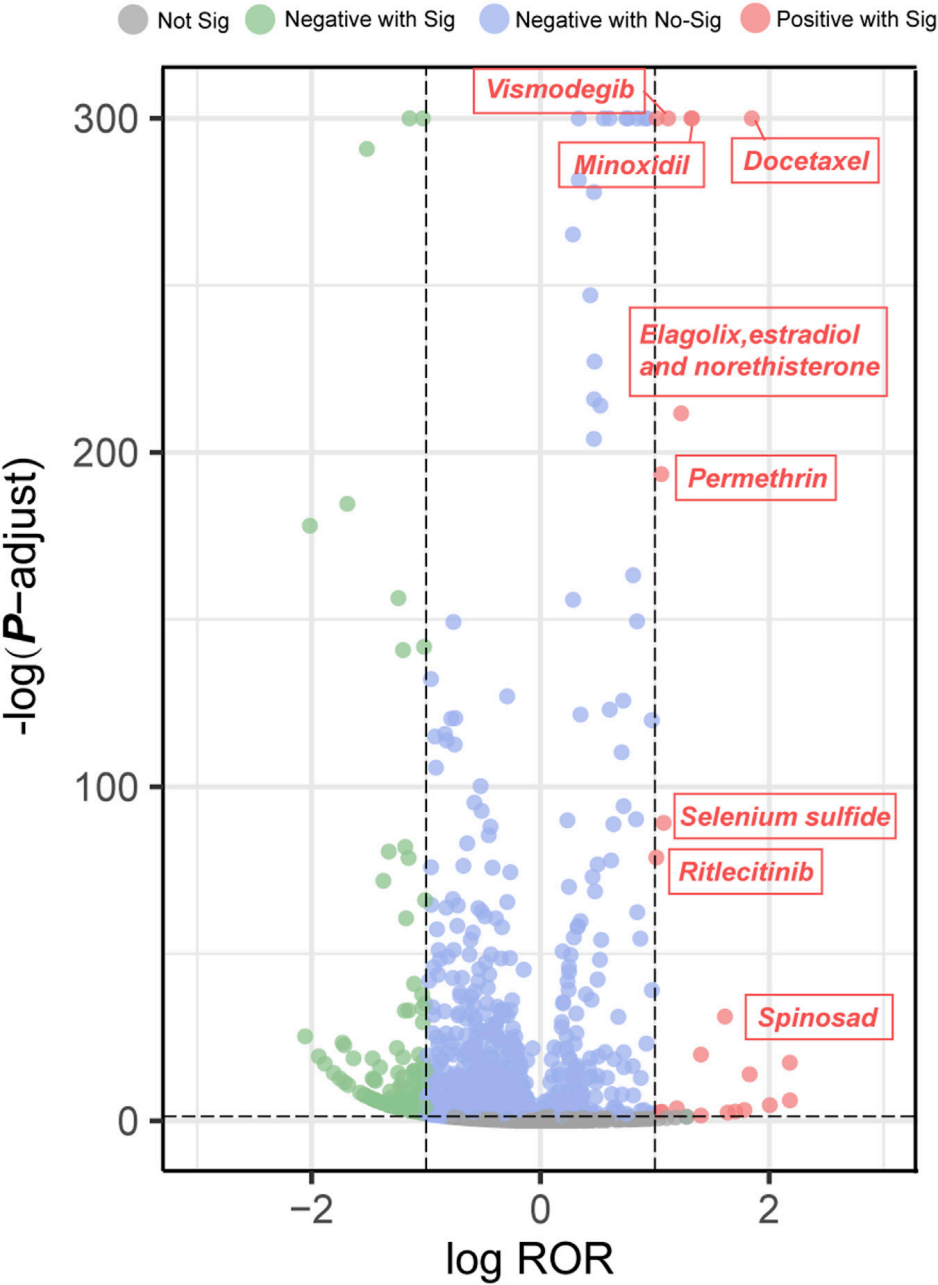
Volcano plot of drug-associated alopecia. Abbreviations: ROR, reporting odds ratio; P-adjust, p-value after Bonferroni correction.

Disproportionality analysis was used to identify drugs with positive signals for alopecia. Firstly, the volcano plot was generated to visualize the relationship between alopecia reports and the suspected drugs (Figure 3). In the plot, the x-axis represents the logarithm of the ROR. A positive value on x-axis suggests that AEs associated with drug-associated alopecia were reported more frequently than other AEs. The y-axis shows the negative logarithm of the *p*-adjust value, which is derived from the *p*-value following Fisher’s exact test and Bonferroni correction. A positive value on the y-axis indicates a highly significant difference. Therefore, drugs located in the upper-right quadrant of the graph exhibited both a strong association signal (high ROR) and statistical significance.

**FIGURE 4:**
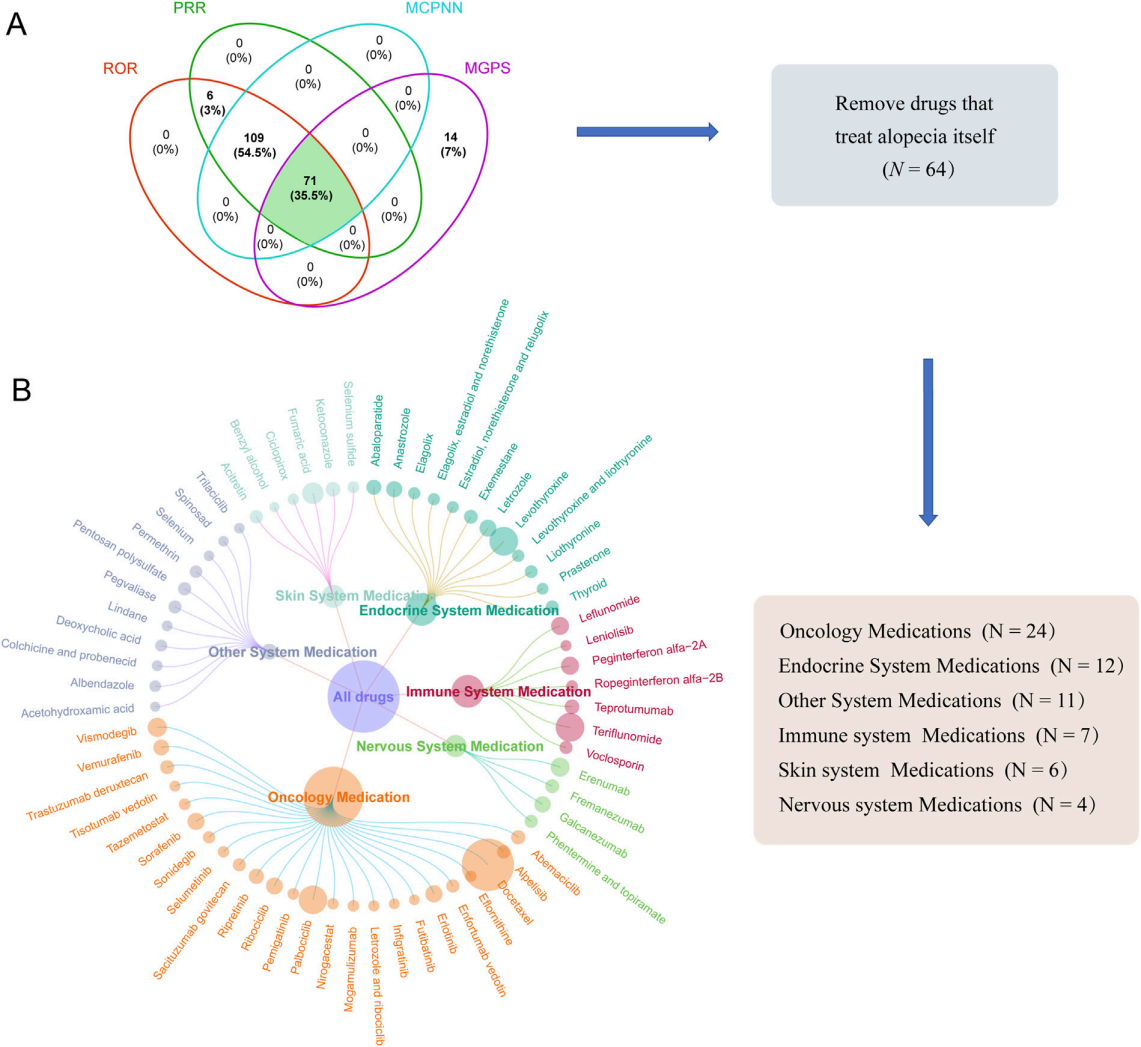
Distribution of drugs with positive signals for drug-associated alopecia. **(A)** Venn diagram of four disproportionality analysis methods. **(B)** Doughnut chart of drugs distribution. The size of each circle represents the number of adverse event reports. Abbreviations: ROR, reporting odds ratio; PRR, proportional reporting ratio; BCPNN, Bayesian confidence propagation neural network; MGPS, Multi-item Gamma Poisson Shrinker.

Subsequently, we analyzed a dataset of 1,545 drugs that reported AEs related to alopecia and identified 71 drugs exhibiting positive signals using all four predefined screening criteria simultaneously (Figure 4A). To focus on drugs where alopecia is likely an unintended adverse effect, we excluded those which are approved or recommended by clinical guidelines for the treatment of alopecia (e.g., AA, AGA) (Supplementary Table S4). Their positive signals were considered potentially confounded by reports of treatment failure or inadequate efficacy. This resulted in 64 drugs for final analysis. Among these, oncology medication constituted the largest proportion (24 drugs, 37.5%), followed by endocrine system medication (12 drugs, 18.8%), immune system medication (7 drugs, 10.9%), skin system medication (6 drugs, 9.4%), nervous system medication (4 drugs, 6.3%), and other therapeutic categories (11 drugs, 17.2%). Figure 4B visualized this distribution with circle sizes proportional to AE report numbers. The top three drugs by AE reports per category were: Oncology - docetaxel, palbociclib, vismodegib; Endocrine system - levothyroxine, letrozole, anastrozole; Immune system - teriflunomide, peginterferon alfa-2a, leflunomide; Skin system - fumaric acid, ketoconazole, acitretin; Nervous system - erenumab, galcanezumab, fremanezumab; Other medications - pentosan polysulfate, pegvaliase, permethrin. Additional details were provided in Figure 4B.

### 3.3 Risk assessment of drugs with positive signals for alopecia

**FIGURE 5:**
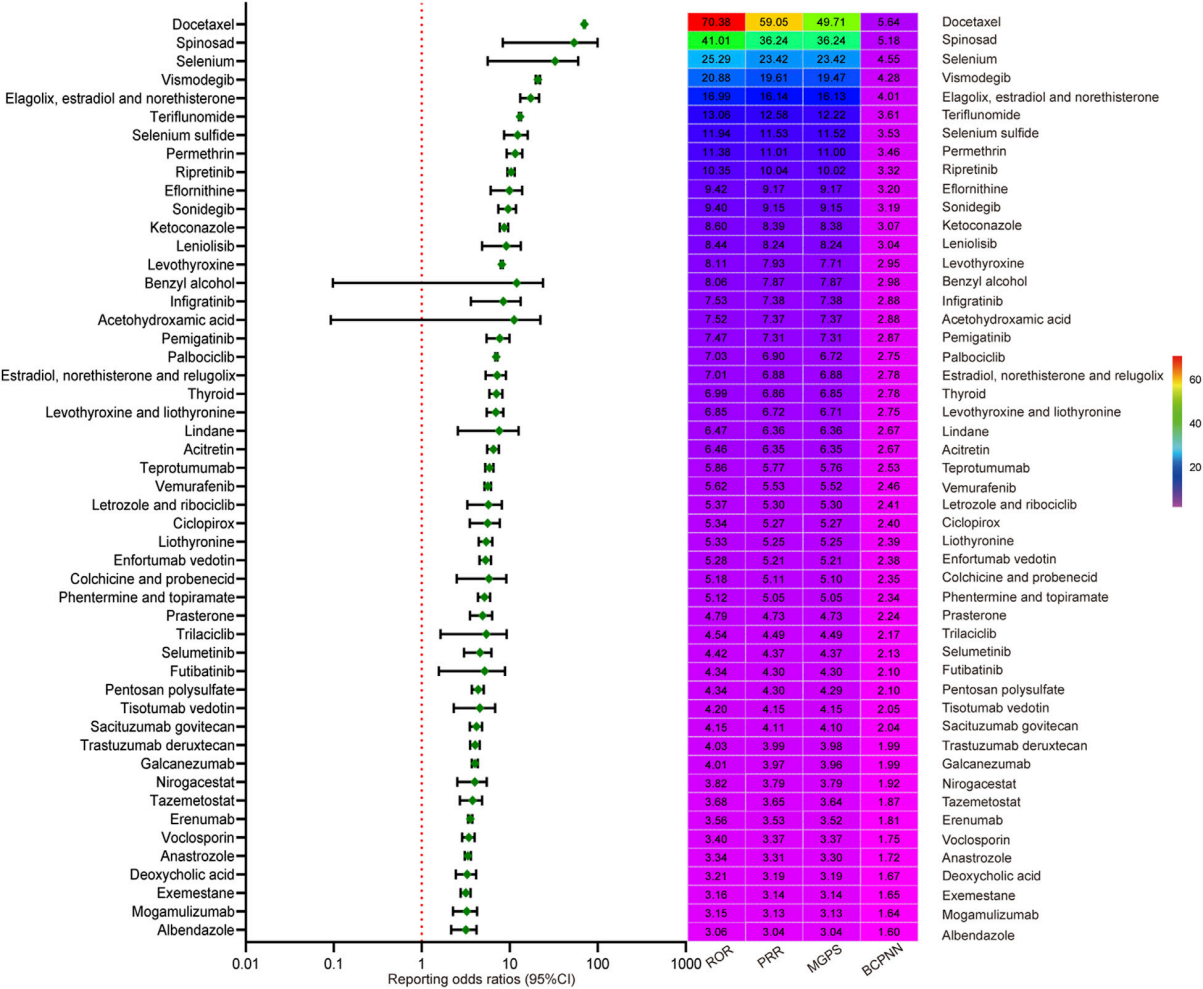
Forest plot and signal value heatmap of top 50 drugs with positive signals from four disproportionality analysis methods. Abbreviations: ROR, reporting odds ratio; PRR, proportional reporting ratio; BCPNN, Bayesian confidence propagation neural network; MGPS, Multi-item Gamma Poisson Shrinker; CI, confidence interval.

The top 50 drugs with positive signals from four disproportionality analyses were presented in Figure 5. Docetaxel showed the highest association strength (ROR = 70.38, 95%CI: 69.43–71.33), followed by Spinosad (ROR = 41.01, 95%CI: 16.12–104.33) and Selenium (ROR = 25.29, 95%CI: 10.15–62.97). Detailed results are available in Table 2 and Figure 5. Subgroup analyses by age and gender further delineated the association profiles. In the age-stratified analysis, 10, 31, 28, and 34 drugs showed significant signals in the <18, 18–44, 45–64, and ≥65-year-old subgroups, respectively. After applying the Bonferroni correction, five additional drugs, anastrozole, tisotumab vedotin, enfortumab vedotin, tazemetostat, and colchicine and probenecid, lost statistical significance in the age-stratified analysis (Supplementary Figure S1). Similarly, in the gender subgroup analysis, 45 and 32 drugs showed positive signals in the subgroups of female and male, respectively. And seven drugs, futibatinib, colchicine and probenecid, infigratinib, acetohydroxamic acid, nirogacestat, liothyronine, and levothyroxine and liothyronine, were no longer significant after Bonferroni correction (Supplementary Figure S2). All Bonferroni-adjusted p-values were presented in the forest plots.

**TABLE 2:** Disproportionality analysis results of drugs with positive signals for drug-associated alopecia.

Drug name	Category	Case reports	ROR (95% CI)	PRR (χ^2^)	IC(IC_025_)	EBGM (EBGM_05_)	*P* Value
Docetaxel	Oncology medication	29,249	70.38 (69.43,71.33)	59.05 (1,405,223)	5.64 (5.61)	5.64 (5.61)	<0.001
Teriflunomide	Immune system medicaiton	5,702	13.06 (12.72,13.42)	12.58 (59,068.2)	3.61 (3.57)	3.61 (3.57)	<0.001
Levothyroxine	Endocrine system medication	5,644	8.11 (7.89,8.33)	7.93 (33,211.8)	2.95 (2.91)	2.95 (2.91)	<0.001
Palbociclib	Oncology medication	5,553	7.03 (6.84,7.22)	6.90 (27,241.4)	2.75 (2.71)	2.75 (2.71)	<0.001
Fumaric acid	Skin system medication	2,109	2.16 (2.07,2.26)	2.15 (1,289.96)	1.10 (1.03)	1.10 (1.03)	<0.001
Vismodegib	Oncology medication	1,328	20.88 (19.75,22.08)	19.61 (23,354.6)	4.28 (4.18)	4.28 (4.18)	<0.001
Erenumab	Nervous system medication	1,287	3.56 (3.37,3.77)	3.53 (2,329.45)	1.81 (1.73)	1.81 (1.73)	<0.001
Peginterferon alfa-2A	Immune system medicaiton	1,050	2.73 (2.57,2.90)	2.71 (1,131.87)	1.43 (1.34)	1.43 (1.34)	<0.001
Leflunomide	Immune system medicaiton	1,014	2.94 (2.76,3.12)	2.92 (1,274.11)	1.54 (1.44)	1.54 (1.44)	<0.001
Erlotinib	Oncology medication	818	2.24 (2.09,2.40)	2.23 (554.34)	1.15 (1.05)	1.15 (1.05)	<0.001
Sorafenib	Oncology medication	808	2.97 (2.77,3.18)	2.95 (1,040.93)	1.56 (1.45)	1.56 (1.45)	<0.001
Letrozole	Endocrine system medication	781	2.94 (2.74,3.16)	2.93 (988.92)	1.54 (1.44)	1.54 (1.44)	<0.001
Ribociclib	Oncology medication	745	2.93 (2.73,3.15)	2.91 (934.38)	1.54 (1.43)	1.54 (1.43)	<0.001
Galcanezumab	Nervous system medication	651	4.01 (3.71,4.33)	3.97 (1,446.40)	1.99 (1.86)	1.99 (1.86)	<0.001
Anastrozole	Endocrine system medication	608	3.34 (3.08,3.61)	3.31 (980.69)	1.72 (1.60)	1.72 (1.60)	<0.001
Vemurafenib	Oncology medication	538	5.62 (5.16,6.12)	5.53 (1997.81)	2.46 (2.33)	2.46 (2.33)	<0.001
Ripretinib	Oncology medication	430	10.35 (9.40,11.39)	10.04 (3,502.70)	3.32 (3.15)	3.32 (3.15)	<0.001
Abaloparatide	Endocrine system medication	397	2.24 (2.03,2.47)	2.23 (270.23)	1.16 (1.01)	1.16 (1.01)	<0.001
Teprotumumab	Immune system medicaiton	351	5.86 (5.28,6.52)	5.77 (1,386.81)	2.53 (2.35)	2.53 (2.35)	<0.001
Ketoconazole	Skin system medication	329	8.60 (7.71,9.60)	8.39 (2,145.81)	3.07 (2.87)	3.07 (2.87)	<0.001
Abemaciclib	Oncology medication	278	2.86 (2.54,3.21)	2.84 (331.61)	1.50 (1.32)	1.50 (1.32)	<0.001
Trastuzumab deruxtecan	Oncology medication	251	4.03 (3.56,4.56)	3.99 (562.88)	1.99 (1.79)	1.99 (1.79)	<0.001
Exemestane	Endocrine system medication	239	3.16 (2.78,3.59)	3.14 (349.11)	1.65 (1.45)	1.65 (1.45)	<0.001
Fremanezumab	Nervous system medication	237	3.00 (2.64,3.41)	2.98 (311.93)	1.57 (1.37)	1.57 (1.37)	<0.001
Alpelisib	Oncology medication	192	2.49 (2.16,2.88)	2.48 (170.28)	1.31 (1.09)	1.31 (1.09)	<0.001
Enfortumab vedotin	Oncology medication	169	5.28 (4.54,6.15)	5.21 (576.50)	2.38 (2.12)	2.38 (2.12)	<0.001
Acitretin	Skin system medication	166	6.46 (5.54,7.54)	6.35 (750.09)	2.67 (2.39)	2.67 (2.39)	<0.001
Pentosan polysulfate	Other medication	160	4.34 (3.72,5.08)	4.30 (405.63)	2.10 (1.84)	2.10 (1.84)	<0.001
Phentermine and topiramate	Nervous system medication	155	5.12 (4.37,6.00)	5.05 (505.27)	2.34 (2.07)	2.34 (2.07)	<0.001
Sacituzumab govitecan	Oncology medication	151	4.15 (3.53,4.87)	4.11 (355.53)	2.04 (1.77)	2.04 (1.77)	<0.001
Voclosporin	Immune system medicaiton	147	3.40 (2.89,4.00)	3.37 (245.65)	1.75 (1.49)	1.75 (1.49)	<0.001
Pegvaliase	Other medication	143	2.79 (2.37,3.29)	2.78 (163.05)	1.47 (1.21)	1.47 (1.21)	<0.001
Thyroid	Endocrine system medication	137	6.99 (5.90,8.28)	6.86 (687.18)	2.78 (2.47)	2.78 (2.47)	<0.001
Liothyronine	Endocrine system medication	125	5.33 (4.46,6.36)	5.25 (431.39)	2.39 (2.08)	2.39 (2.08)	<0.001
Permethrin	Other medication	98	11.38 (9.30,13.93)	11.01 (893.98)	3.46 (3.02)	3.46 (3.02)	<0.001
Levothyroxine and liothyronine	Endocrine system medication	85	6.85 (5.52,8.49)	6.72 (414.76)	2.75 (2.34)	2.75 (2.34)	<0.001
Elagolix	Endocrine system medication	77	2.72 (2.17,3.40)	2.70 (82.97)	1.44 (1.08)	1.44 (1.08)	<0.001
Sonidegib	Oncology medication	76	9.40 (7.48,11.82)	9.15 (553.45)	3.19 (2.71)	3.19 (2.71)	<0.001
Elagolix, estradiol and norethisterone	Endocrine system medication	69	16.99 (13.33,21.65)	16.14 (982.74)	4.01 (3.37)	4.01 (3.37)	<0.001
Deoxycholic acid	Other medication	57	3.21 (2.47,4.17)	3.19 (85.89)	1.67 (1.24)	1.67 (1.24)	<0.001
Estradiol, norethisterone and relugolix	Endocrine system medication	57	7.01 (5.39,9.12)	6.88 (287.13)	2.78 (2.26)	2.78 (2.26)	<0.001
Ropeginterferon alfa-2B	Immune system medicaiton	53	2.74 (2.09,3.59)	2.73 (58.07)	1.45 (1.01)	1.45 (1.01)	<0.001
Prasterone	Endocrine system medication	48	4.79 (3.60,6.37)	4.73 (141.69)	2.24 (1.72)	2.24 (1.72)	<0.001
Tazemetostat	Oncology medication	48	3.68 (2.77,4.89)	3.65 (92.44)	1.87 (1.37)	1.87 (1.37)	<0.001
Pemigatinib	Oncology medication	46	7.47 (5.57,10.00)	7.31 (251.34)	2.87 (2.26)	2.87 (2.26)	<0.001
Selenium sulfide	Skin system medication	43	11.94 (8.80,16.19)	11.53 (414.61)	3.53 (2.77)	3.53 (2.77)	<0.001
Mogamulizumab	Oncology medication	41	3.15 (2.32,4.29)	3.13 (59.54)	1.64 (1.13)	1.64 (1.13)	<0.001
Albendazole	Other medication	37	3.06 (2.21,4.23)	3.04 (50.77)	1.60 (1.06)	1.60 (1.06)	<0.001
Selumetinib	Oncology medication	32	4.42 (3.12,6.26)	4.37 (83.40)	2.13 (1.48)	2.13 (1.48)	<0.001
Nirogacestat	Oncology medication	28	3.82 (2.63,5.55)	3.79 (57.57)	1.92 (1.25)	1.92 (1.25)	<0.001
Ciclopirox	Skin system medication	27	5.34 (3.65,7.82)	5.27 (93.70)	2.40 (1.64)	2.40 (1.64)	<0.001
Eflornithine	Oncology medication	25	9.42 (6.33,14.03)	9.17 (182.57)	3.20 (2.23)	3.20 (2.23)	<0.001
Letrozole and ribociclib	Oncology medication	21	5.37 (3.49,8.27)	5.30 (73.44)	2.41 (1.53)	2.41 (1.53)	<0.001
Leniolisib	Immune system medicaiton	17	8.44 (5.21,13.67)	8.24 (108.52)	3.04 (1.86)	3.04 (1.86)	<0.001
Tisotumab vedotin	Oncology medication	15	4.20 (2.52,6.98)	4.15 (36.00)	2.05 (1.07)	2.05 (1.07)	<0.001
Colchicine and probenecid	Other medication	11	5.18 (2.85,9.39)	5.11 (36.43)	2.35 (1.09)	2.35 (1.09)	<0.001
Infigratinib	Oncology medication	11	7.53 (4.14,13.71)	7.38 (60.83)	2.88 (1.42)	2.88 (1.42)	<0.001
Lindane	Other medication	8	6.47 (3.21,13.04)	6.36 (36.25)	2.67 (1.02)	2.67 (1.02)	<0.001
Futibatinib	Oncology medication	7	4.34 (2.06,9.16)	4.30 (17.76)	2.10 (0.58)	2.10 (0.58)	<0.001
Trilaciclib	Other medication	7	4.54 (2.15,9.57)	4.49 (19.03)	2.17 (0.61)	2.17 (0.61)	<0.001
Selenium	Other medication	5	25.29 (10.15,62.97)	23.42 (107.66)	4.55 (1.07)	4.55 (1.07)	<0.001
Spinosad	Other medication	5	41.01 (16.12,104.33)	36.24 (171.91)	5.18 (1.14)	5.18 (1.14)	<0.001
Acetohydroxamic acid	Other medication	3	7.52 (2.39,23.65)	7.37 (16.56)	2.88 (0.04)	2.88 (0.04)	0.001
Benzyl alcohol	Skin system medication	3	8.06 (2.56,25.35)	7.87 (18.06)	2.98 (0.06)	2.98 (0.06)	<0.001

ROR, reporting odds ratio; PRR, proportional reporting ratio; IC, information component; IC_025_, lower limit of the 95% CI, of the IC; EBGM_05_, empirical Bayesian geometric mean lower 95% CI, for the posterior distribution.

### 3.4 Stratified evaluation based on reporting frequency, drug category, risk level, and drug-induced time

**FIGURE 6:**
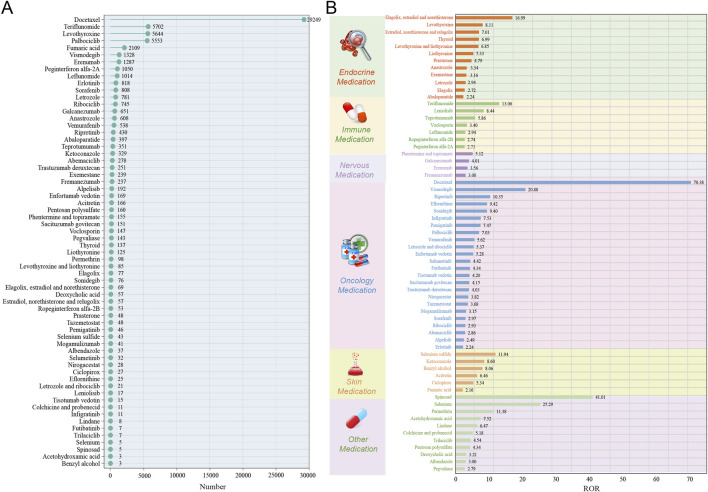
Reporting frequency and therapeutic categorization of drugs with positive alopecia signals. **(A)** Drugs ranked by the number of adverse event reports. **(B)** Drugs categorized by therapeutic class and ranked by ROR within each category.Abbreviations ROR, reporting odds ratio.

**FIGURE 7:**
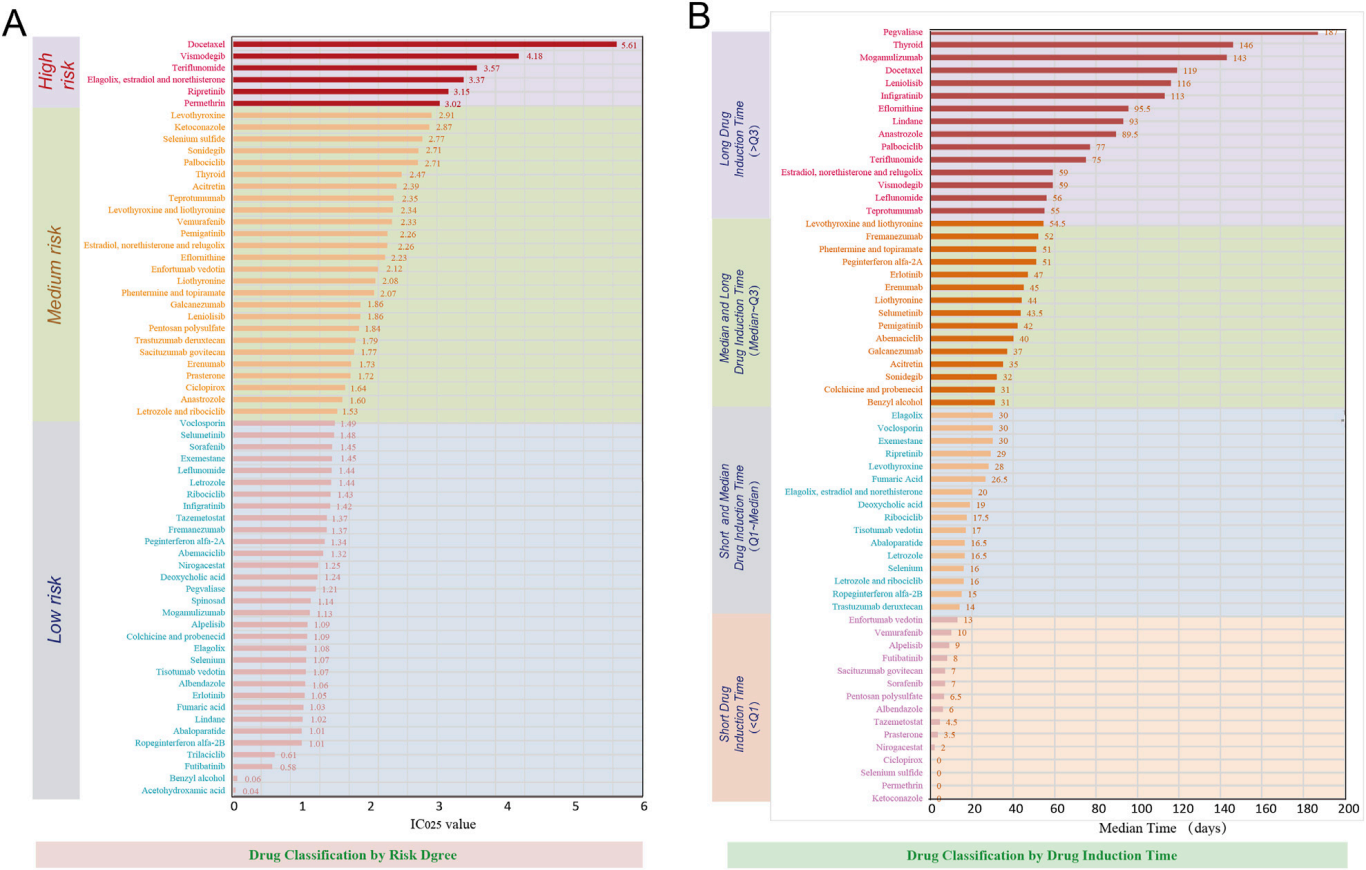
Risk level and onset time distribution of drugs with positive alopecia signals. **(A)** Drugs categorized by risk level based on IC_025_ values. **(B)** Drugs ranked by median drug-induced onset time. Abbreviations: IC_025_, lower limit of the 95% CI of the information component.

The drugs sorted by the number of reports were presented in Figure 6A. During the study period, the top four drugs reporting the highest number of alopecia-associated AEs were docetaxel, teriflunomide, levothyroxine and palbociclib, each exceeding 5,000 reports. For each drug category, the top three drugs by ROR values for endocrine medication were estradiol, norethisterone and relugolix (ROR = 16.99, 95%CI: 13.33–21.65), levothyroxine (ROR = 8.11, 95%CI: 7.89–8.33), elagolix, estradiol and norethisterone (ROR = 7.01, 95%CI: 5.39–9.12); for immune system medication, the top three drugs in ROR values were teriflunomide (ROR = 13.06, 95%CI: 12.72–13.42), leniolisib (ROR = 8.44, 95%CI: 5.21–13.67), and teprotumumab (ROR = 5.86, 95%CI: 5.28–6.52); for nervous system medication, the top three drugs in ROR values were phentermine and topiramate (ROR = 5.12, 95%CI: 4.37–6.00), galcanezumab (ROR = 4.01, 95%CI: 3.71–4.33), and erenumab (ROR = 3.56, 95%CI: 3.37–3.77); for oncology medication, the top three drugs in ROR values were docetaxel (ROR = 70.38, 95%CI: 69.43–71.33), vismodegib (ROR = 20.88, 95%CI: 19.75–22.08), and ripretinib (ROR = 10.35, 95%CI: 9.40–11.39); for skin system medication, the top three drugs in ROR values were selenium sulfide (ROR = 11.94, 95%CI: 8.80–16.19), ketoconazole (ROR = 8.60, 95%CI: 7.71–9.60), and benzyl alcohol (ROR = 8.06, 95%CI: 2.56–25.35); for other medication, the top three drugs in ROR values were Spinosad (ROR = 41.01, 95%CI: 16.12–104.33), selenium (ROR = 25.29, 95%CI: 10.15–62.97), and permethrin (ROR = 11.38, 95%CI: 9.30–13.93). More details were provided in Figure 6B. Based on the IC_025_ values, the 64 drugs were classified into high-risk (IC_025_ > 3), medium-risk (1.5 < IC_025_ ≤ 3), and low-risk (IC_025_ ≤ 1.5) categories (Wu et al., 2025). This resulted in 6 high-risk drugs (9.38%), 26 medium-risk drugs (40.63%), and 32 low-risk drugs (50.00%). Docetaxel (IC_025_ = 5.61), vismodegib (IC_025_ = 4.18), and teriflunomide (IC_025_ = 3.57) represented the top three highest-risk drugs (Figure 7A). Additionally, drugs were categorized by drug-induced onset time duration, segmented by quartiles. The top three drugs with the longest median drug-induced onset time were pegvaliase (median time = 187 days), thyroid (median time = 146 days), and mogamulizumab (median time = 143 days) (Figure 7B).

### 3.5 Proportional distribution of drugs at different PT levels

To further summarize the overall drug characteristics of DIA, we integrated the positive/negative distribution of ADR signal for drugs at the PT level and the corresponding drug class distributions (anatomical therapeutic chemical (ATC) classification system) (Supplementary Figure S3). Overall, the number of drugs with positive ADR signals was lower than those with negative ADR signals in all groups. Notably, antineoplastic agents (L01) and immunosuppressants (L04) exhibited the highest proportion of positive signals at most PT levels, except for non-scarring alopecia, injection site alopecia, and application site alopecia. No positive signals were observed for loose anagen syndrome, and no drugs were reported to induce seborrhoeic alopecia.

**FIGURE 8:**
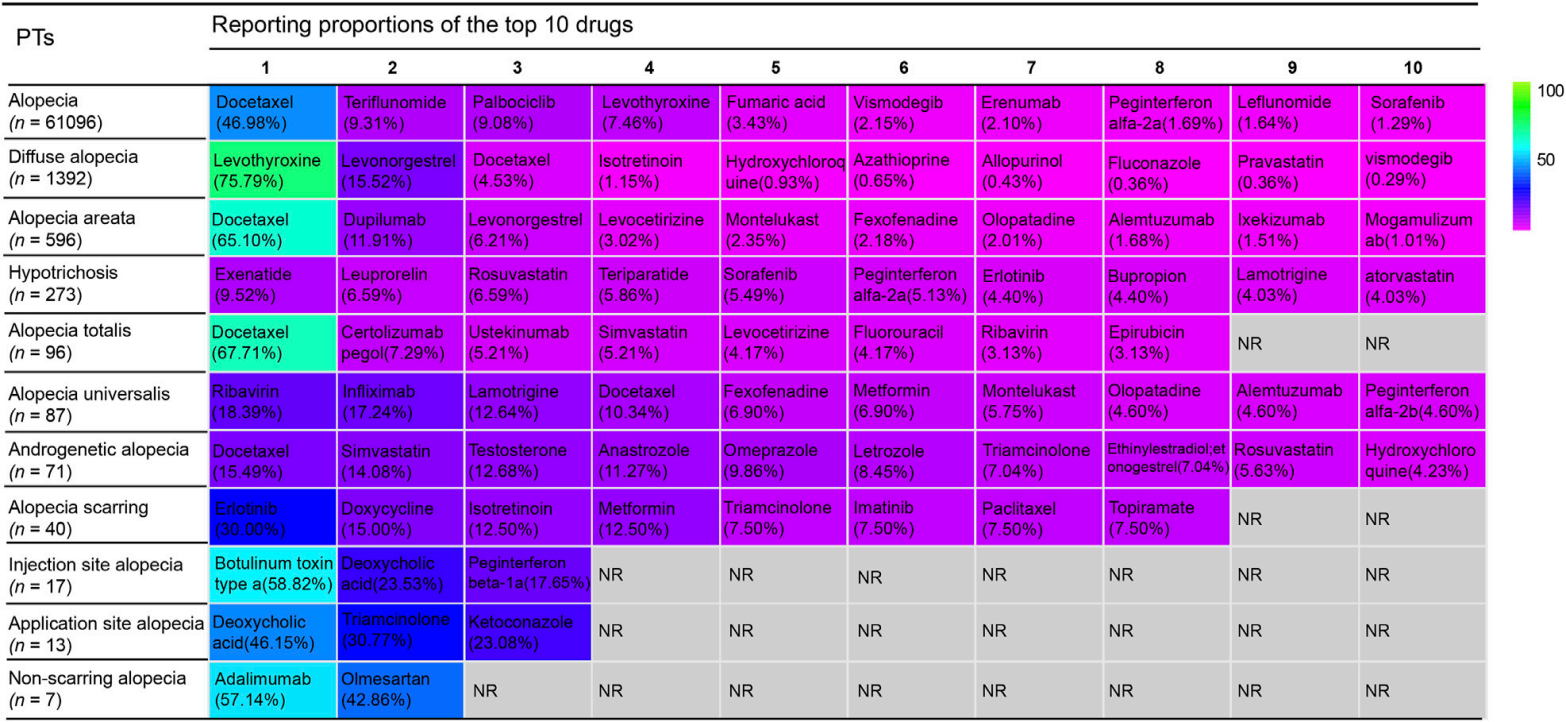
The distribution of top 10 drugs at different performed terms.

Figure 8 showed the top 10 drugs with the highest reporting proportions at the PT level. Docetaxel was the most frequently reported drug for alopecia, AA, alopecia totalis, and AGA, with reporting proportions of 46.98%, 65.10%, 67.71%, and 15.49%, respectively. Levothyroxine showed the highest reporting proportion for diffuse alopecia (75.79%). Exenatide (9.52%), ribavirin (18.39%), and erlotinib (30.00%) accounted for the highest proportions of hypotrichosis, alopecia universalis, and alopecia scarring, respectively. Only botulinum toxin type a, deoxycholic acid, and peginterferon beta-1a were associated with injection site alopecia. Deoxycholic acid, triamcinolone, and ketoconazole were the only drugs reported to cause application site alopecia, while only adalimumab and olmesartan reported non-scarring alopecia.

### 3.6 Comparison of drug-induced onset time among gender and drug category

**FIGURE 9:**
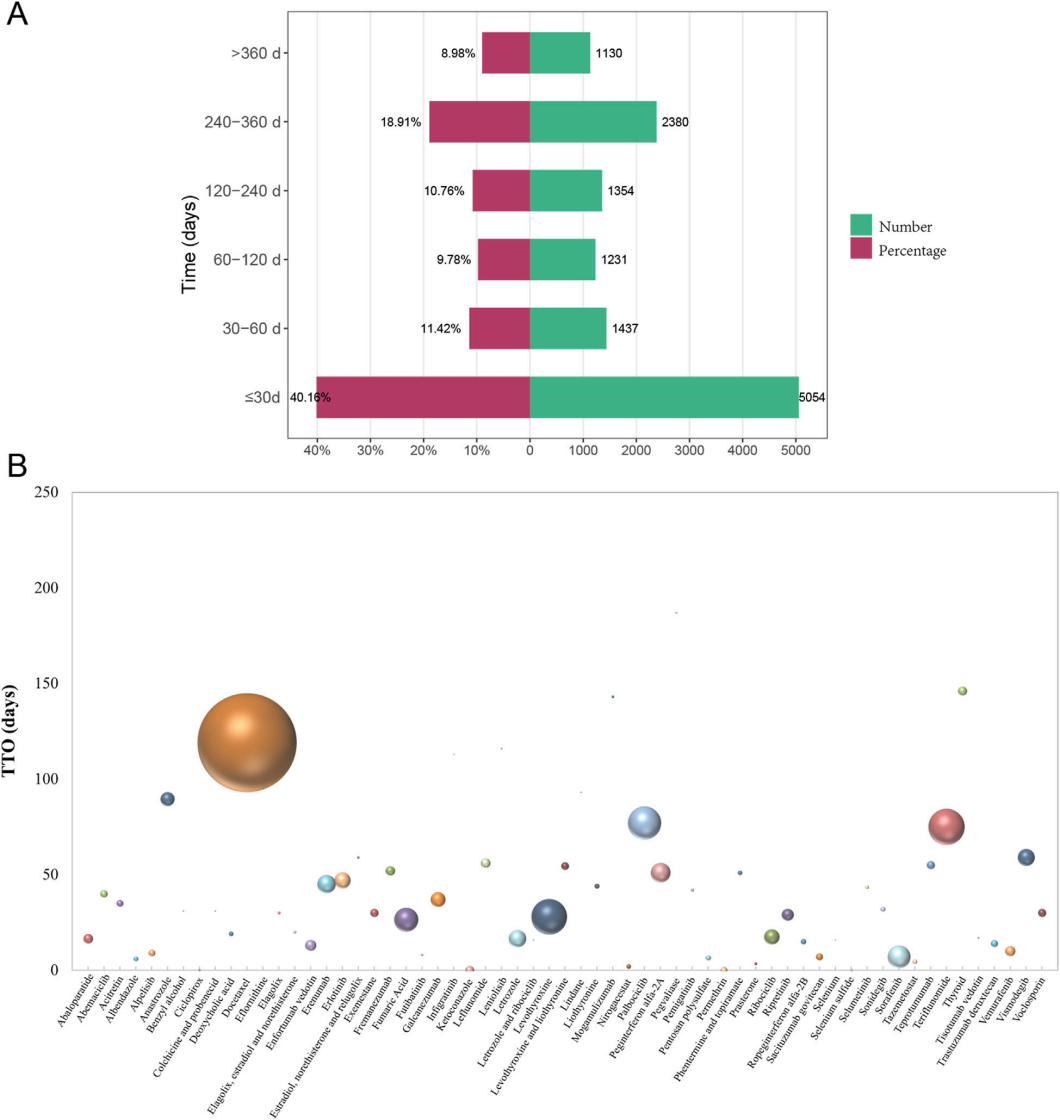
**(A)** The time distribution of drug-induced alopecia onset time for drugs with positive signals. **(B)** The TTO bubbles distribution diagram for each drug. Abbreviations: TTO, time to onset.

Figure 9A illustrated the time distribution of drug-induced onset time for drugs with positive signals. Among them, 40.16% (5,054 reports) of cases induced alopecia AEs within 30 days. And there were 2,380 reports documented onset times of alopecia between 240 and 360 days Figure 9B presented the TTO bubble plot for individual drugs, where circle size corresponded to report frequency. Among them, docetaxel had the highest number of reports (median time = 119 days). Pegvaliase showed the longest median onset time, at 187 days, although only 3 reports have reporting time data. The induction time of most drugs was within 15–50 days. See Table 3 for further details. We further analyzed gender differences in TTO and found that only docetaxel use resulted in a significantly shorter latency in males compared to females (*P* < 0.001, Supplementary Table S5).

**TABLE 3:** Time to onset for drug-induced alopecia by different drugs.

Drug name	Case reports (n)	Median (days)	Q1 (days)	Q3 (days)
Pegvaliase	3	187	176	530
Thyroid	59	146	4	1,130
Mogamulizumab	5	143	0	952
Docetaxel	7,038	119	27	283
Leniolisib	3	116	76	289
Infigratinib	1	113	113	113
Eflornithine	4	95.5	13.5	812.5
Lindane	1	93	93	93
Anastrozole	140	89.5	13	365
Palbociclib	791	77	13	293
Teriflunomide	941	75	17	215
Vismodegib	206	59	14	156
Estradiol, norethisterone and relugolix	4	59	18.5	115.5
Leflunomide	67	56	9	123
Teprotumumab	46	55	7	151
Levothyroxine and liothyronine	42	54.5	7	118
Fremanezumab	63	52	3	148
Peginterferon alfa-2A	275	51	7	121
Phentermine and topiramate	15	51	8	212
Erlotinib	189	47	7	143
Erenumab	242	45	5	122
Liothyronine	17	44	0	196
Selumetinib	10	43.5	10	137
Pemigatinib	7	42	0	102
Abemaciclib	39	40	8	80
Galcanezumab	159	37	7	100
Acitretin	34	35	0	106
Sonidegib	17	32	17	200
Benzyl alcohol	1	31	31	31
Colchicine and probenecid	1	31	31	31
Exemestane	49	30	0	334
Voclosporin	48	30	1.5	82.5
Elagolix	6	30	3	39
Ripretinib	102	29	4	134
Levothyroxine	907	28	2	108
Fumaric acid	414	26.5	0	136
Elagolix, estradiol and norethisterone	7	20	4	61
Deoxycholic acid	15	19	1	28
Ribociclib	168	17.5	3	120
Tisotumab vedotin	2	17	17	17
Letrozole	218	16.5	5	61
Abaloparatide	66	16.5	0	62
Letrozole and ribociclib	2	16	0	32
Selenium	1	16	16	16
Ropeginterferon alfa-2B	21	15	0	75
Trastuzumab deruxtecan	36	14	0	154.5
Enfortumab vedotin	89	13	6	20
Vemurafenib	74	10	4	27
Alpelisib	33	9	0	64
Futibatinib	3	8	0	35
Sorafenib	370	7	2	18
Sacituzumab govitecan	35	7	2	22
Pentosan polysulfate	20	6.5	0	648
Albendazole	17	6	2	19
Tazemetostat	16	4.5	0	57.5
Prasterone	6	3.5	0	92
Nirogacestat	15	2	0	12
Ketoconazole	56	0	0	0
Permethrin	32	0	0	1.5
Selenium sulfide	6	0	0	28
Ciclopirox	3	0	0	43

**FIGURE 10:**
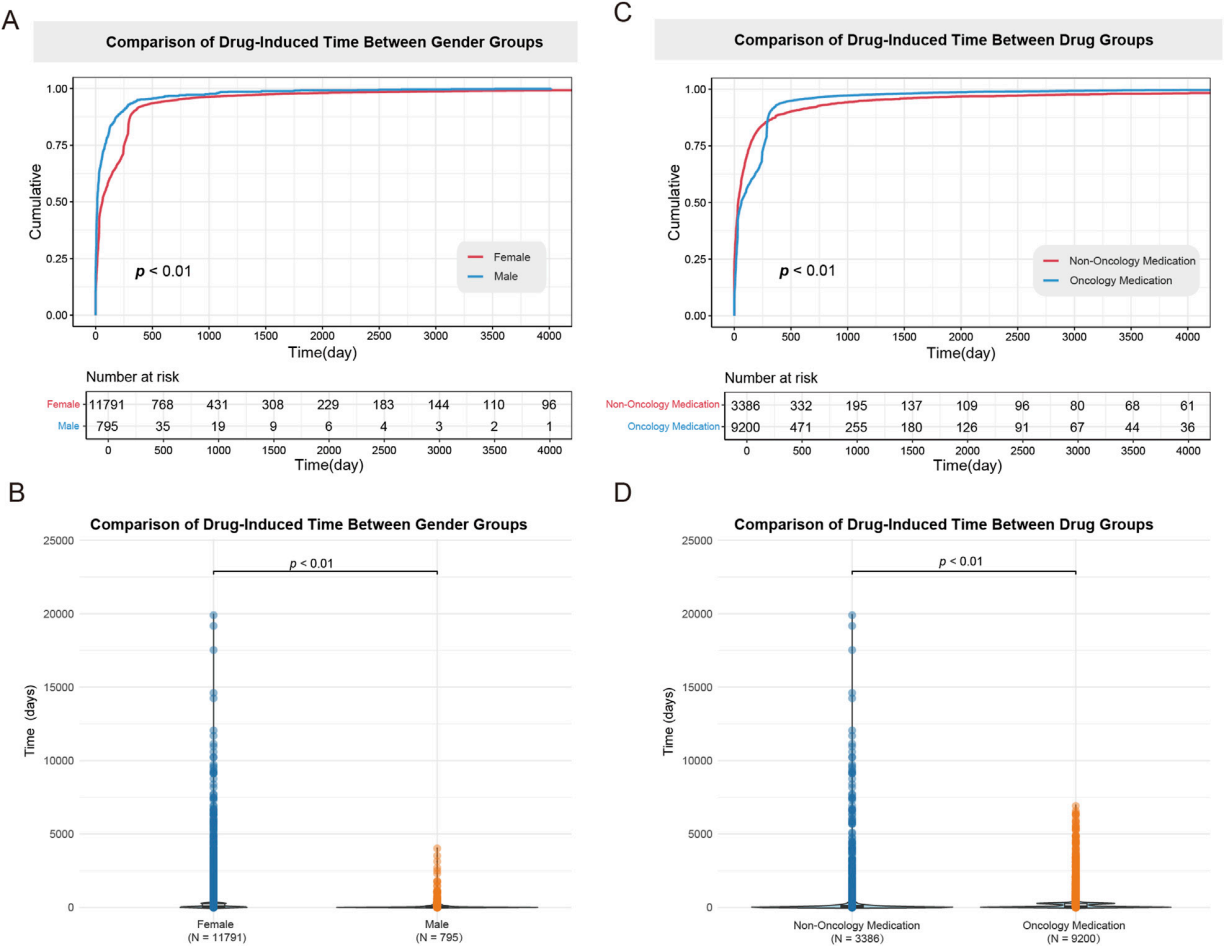
Comparison of time-to-onset for drug-induced alopecia by gender and drug categories. **(A)** Cumulative risk curve for drug-induced alopecia onset times between female and male groups. **(B)** Violin plot for drug-induced alopecia onset times between female and male groups. The results illustrated that males have significantly shorter drug induction times compared to females (*P* < 0.01). **(C)** Cumulative risk curve for drug-induced alopecia onset times between oncology and non-oncology medications. **(D)** Violin plot for drug-induced alopecia onset times between oncology and non-oncology medications. The results illustrated that oncology medications have significantly shorter drug induction times compared to non-oncology medications (*P* < 0.01).

The cumulative curves and violin plots were used to evaluate the differences in drug-induced time between genders and different category of drugs, as shown in Figure 10. The results illustrated that males experienced a significantly shorter drug-induced onset time compared to females (mean days, 108 days vs. 236 days, respectively; *P* < 0.001). Furthermore, a comparison between oncology and non-oncology medications demonstrated a significant difference, with oncology medications inducing a notably shorter onset time than non-oncology medications (mean time, 198 days vs. 308 days, respectively; *P* < 0.001).

### 3.7 The relationship between medications and alopecia

**FIGURE 11:**
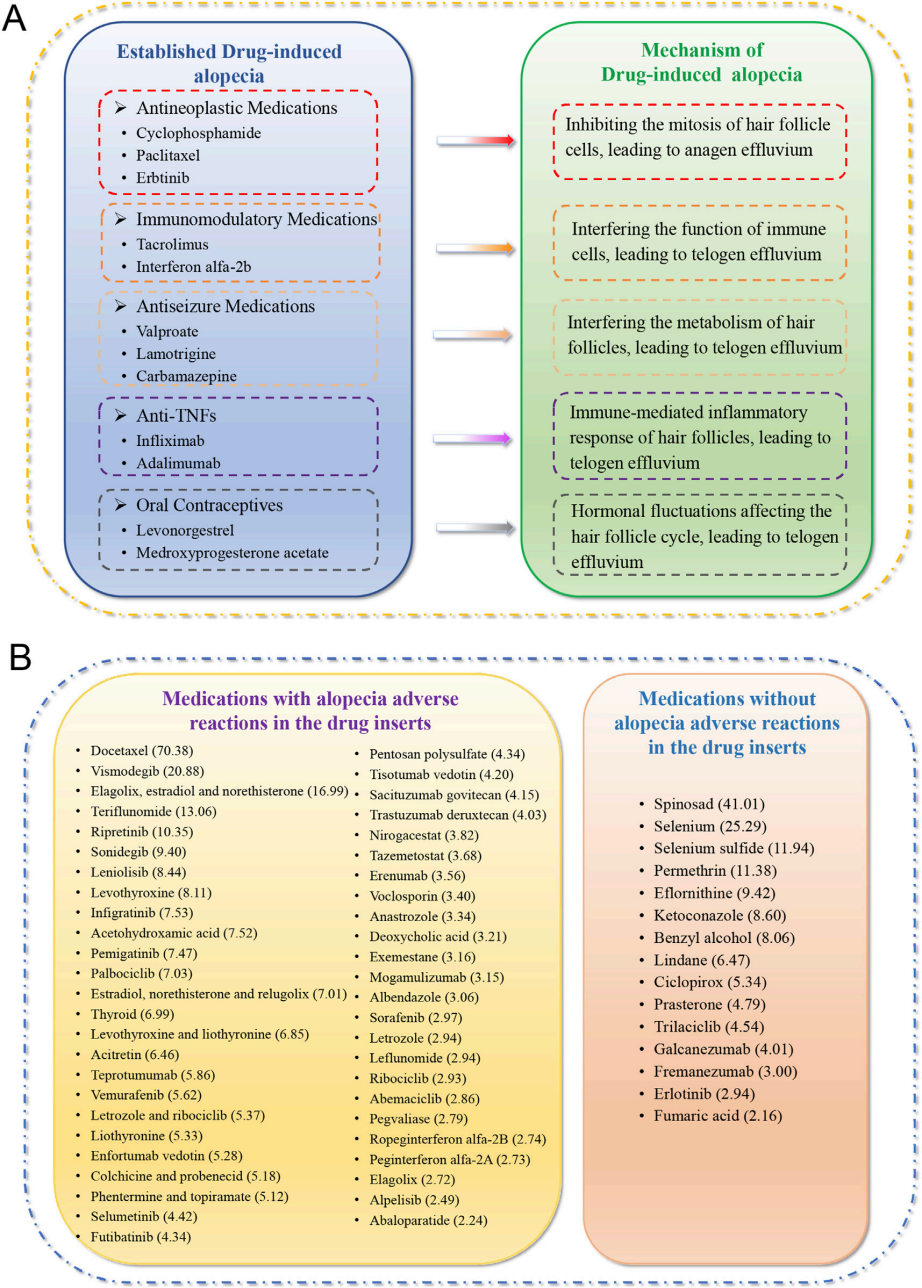
**(A)** The five common drug categories and representative drugs that are established or strongly suspected to be associated with alopecia based on existing literature. **(B)** Drugs with positive signals stratified by presence of alopecia in the adverse reactions section of drug inserts. Values in parentheses represent the reporting odds ratio (ROR).

Based on reports from PubMed and Web of Science, medications with well-established associations with DIA are primarily concentrated in five major therapeutic classes: antineoplastic agents (e.g., cyclophosphamide, paclitaxel), immunomodulators (e.g., tacrolimus, interferon alfa-2b), antiseizure medications (e.g., valproate, carbamazepine), anti-TNF biologics (e.g., infliximab, adalimumab), and hormonal agents (e.g., levonorgestrel-containing contraceptives). The mechanisms underlying DIA vary among these categories. Antineoplastic medications primarily inhibit the mitosis of hair follicle cells, leading to anagen effluvium. Immunomodulatory medications may disrupt immune cell function, resulting in telogen effluvium. Antiseizure medications, anti-TNFs, and oral contraceptives may cause telogen effluvium through metabolic interference in hair follicles, follicle-targeted inflammatory responses, and hormonal disruption of the follicle cycle, respectively (Figure 11A). By comparing with the United States prescribing information, we found that alopecia was documented in the labels of 49 drugs (76.6%) among the 64 drugs with positive signals. In contrast, 15 drugs (23.4%), such as erlotinib, ketoconazole, fremanezumab, galcanezumab, lacked this adverse reaction documentation in their official labels, despite showing significant signals (Figure 11B).

## 4 Discussion

Our large-scale pharmacovigilance study utilized real-world FAERS data to systematically identify and characterize signals of drug-associated alopecia using disproportionality analysis. We identified 64 drugs with significant alopecia signals, revealing distinct risk profiles across therapeutic categories, demographic groups, and latency periods. By quantifying these signals at a population level, our findings expand the current understanding of suspected DIA and provide clinically actionable insights. Furthermore, they highlight specific drugs that may warrant updates to their package inserts based on accumulated real-world evidence.

In terms of patient demographics, reports were predominantly from females (76.82%), peaking in the 50–65 age range. This likely reflects both a higher prevalence of polypharmacy for chronic conditions in this group and a greater perceived impact of hair loss. These findings are consistent with established evidence that women, particularly during hormonal transition periods like perimenopause, experience higher rates of DIA (Mirmirani, 2013). Interestingly, despite higher reporting rates by women, most signaled drugs showed higher relative alopecia risk in men, suggesting potential under-reporting by males or greater biological susceptibility (Supplementary Figure S4). This paradox may be explained by heightened female vigilance regarding hair changes and a greater likelihood to seek medical advice and report events, alongside physiological factors such as higher androgen sensitivity in males potentially increasing their susceptibility (Cash et al., 1993; Trüeb, 2002). Addressing this may require raising awareness among male patients. Furthermore, significant alopecia incidence occurred in older adults (≥65 years), consistent with known elevated ADR risks from age-related pharmacokinetic changes and polypharmacy (Hadia et al., 2022; Ngcobo, 2025). In elderly patients, reduced drug distribution volumes can lead to higher drug concentrations, increasing the risk of adverse events, particularly for drugs with a narrow therapeutic index. This necessitates vigilant monitoring and potential dose adjustments in this population.

Most alopecia reports were submitted by consumers (55.88%), suggesting patients perceive this as a greater concern than healthcare providers. Regarding administration routes, oral medications accounted for the most alopecia reports, followed by subcutaneous and intravenous drugs. This pattern likely reflects the widespread use of oral drugs for chronic conditions, resulting in a large at-risk population. The notable proportion of subcutaneous reports likely stems from the high mechanistic potency of biologics and targeted therapies to disrupt hair follicles. In contrast, intravenous drugs, though potent, are used for shorter durations, leading to fewer reports. Understanding these differences is essential for clinicians to monitor the safety of treatments effectively.

Oncology drugs constituted the largest proportion of high-risk medications for alopecia (37.5%), with docetaxel exhibiting the strongest association (ROR = 70.38). This is aligned with the well-established risk of chemotherapy-induced alopecia (CIA), primarily through anagen effluvium (Trüeb, 2010). Notably, targeted agents like vismodegib and palbociclib also showed prominent signals, suggesting that non-cytotoxic mechanisms, specifically through inhibition of hedgehog signaling or CDK4/6, may similarly disrupt follicular cycling, leading to anagen effluvium. While CIA is usually reversible, (Kinoshita et al., 2019), persistent, or permanent CIA (pCIA) can occur, with taxanes like docetaxel carrying a particularly high risk (Perez et al., 2024). Although no definitive cure for pCIA exists, treatment options such as minoxidil, photobiomodulation, and platelet-rich plasma injections are being explored (Wikramanayake et al., 2023). Scalp cooling remains the primary FDA-approved preventive measure, reducing the relative risk of significant alopecia by over 40% (Rugo and Voigt, 2018; Yin et al., 2023).

Endocrine medications, such as levothyroxine and anastrozole, also featured prominently in the list of drugs associated with alopecia. Levothyroxine may cause hair thinning via effects on thyroid hormone levels, which are crucial for follicle function (van Beek et al., 2008). Notably, thyroid dysfunction itself (encompassing hypothyroidism, hyperthyroidism, and parathyroid disorders) is a well-established contributor to alopecia (Vincent and Yogiraj, 2013). Anastrozole, an aromatase inhibitor for breast cancer treatment, likely induces alopecia through estrogen depletion, potentially by shortening the anagen phase, increasing shedding (telogen effluvium), or contributing to female pattern hair loss (Karatas et al., 2016). Interestingly, apart from interferon alpha, teriflunomide, leniolisib, and teprotumumab, other immunosuppressive drugs were also identified as notable contributors in this study. This highlights the growing recognition of the potential for immunomodulatory therapies to cause alopecia. Migraine medication-calcitonin gene-related peptide (CGRP) inhibitors, such as erenumab (*n* = 1,287, ROR = 3.56), galcanezumab (*n* = 651, ROR = 4.01), and fremanezumab (*n* = 237, ROR = 3.00), also exhibited higher risk signals with more frequent reports. This is consistent with previous reports that CGRP inhibitors can cause alopecia ADR (Woods, 2022; Ruiz et al., 2023), suggesting that drug regulatory agencies should issue warnings and revise the drug label for this issue.

The observed bimodal distribution of TTO for DIA, with a substantial proportion of cases (40.2%) occurring within 30 days and a later peak (13.1%) manifesting at 240–360 days, strongly reflects distinct underlying pathophysiological mechanisms. The early-onset cases (≤30 days) are highly consistent with anagen effluvium. This is characterized by the acute, direct cytotoxicity of medications, most notably antineoplastic agents (e.g., docetaxel) and kinase inhibitors (e.g., sorafenib, vemurafenib). These agents rapidly disrupt the intense mitotic activity of hair matrix keratinocytes during the anagen (growth) phase, leading to abrupt hair loss within days to weeks of drug exposure (Paus et al., 2013). This pathophysiology directly supports our finding of a significantly shorter mean TTO for oncology drugs compared to non-oncology agents (*P* < 0.001). Conversely, the delayed-onset cases (typically ≥90 days and extending beyond 240 days) align with the pattern of telogen effluvium. This form of hair loss is associated with drugs that subtly disrupt the normal hair cycle over a prolonged period, such as endocrine medications (e.g., thyroid agents) and immunomodulatory drugs (e.g., teriflunomide). These drugs may gradually alter hormonal equilibrium or follicular cycling, precipitating a premature and synchronized transition of numerous hair follicles from anagen to the telogen (resting/shedding) phase. The resulting hair loss becomes clinically apparent only after the 2–3 months latency period of the telogen phase, explaining the observed longer TTO (Chien Yin et al., 2021). Consequently, clinical management can reference the agent-specific TTO data: Patients initiating rapid-onset drugs (median ≤15 days) require education on vigilant monitoring within the first 2 weeks for signs like increased shedding on brushes/pillows, while those on delayed-onset agents (median >100 days) need extended surveillance (≥6 months) and counseling to prevent misattribution of late hair loss to aging or stress.

A critical finding was the significant sex-based disparity in DIA latency, with a markedly shorter onset in males (108 days) versus females (236 days). This aligned with established biological differences (Trüeb, 2002; Thornton, 2013). Males exhibit higher androgen sensitivity, where elevated dihydrotestosterone levels accelerate follicular miniaturization and amplify drug-triggered anagen-to-telogen transition. Conversely, estrogen’s protective role in prolonging anagen phase may delay DIA manifestation in females. Notably, while this robust population-level trend was statistically significant only for docetaxel in our drug-specific analyses (*P* < 0.001), the observed discrepancy can be attributed to the combined effects of statistical power and the characteristics of data within spontaneous reporting systems. The population-level analysis benefits from a large aggregated sample, whereas drug-specific comparisons are often underpowered due to limited TTO data for individual agents, failing to reach statistical significance despite a consistent directional trend. Consequently, the finding for docetaxel confirms the general trend is detectable at the individual drug level when data are sufficient. Beyond biological mechanisms, clinical and reporting factors contribute to this disparity (Thornton, 2013). Females more frequently use chronic therapies associated with delayed alopecia (e.g., hormonal agents, antidepressants), and often seek treatment in the hair loss process, typically leading to delayed reporting. Conversely, males report earlier due to visible patterning (e.g., frontal recession) and reduced attribution bias. This underscores the need for sex-specific monitoring protocols in clinical practice. Males should maintain vigilance within 30–60 days for high-risk agents like taxanes and retinoids, while females require extended surveillance of at least 3 months for endocrine or immunomodulatory drugs.

Our analysis further revealed an apparent gap between real-world evidence and official documentation, as 23.4% of drugs with significant alopecia signals did not list alopecia as an adverse reaction in their U.S. prescribing information. For instance, CGRP inhibitors (fremanezumab, galcanezumab), increasingly prescribed for migraine prevention, demonstrated consistent signals across multiple disproportionality metrics, suggesting an under-recognized risk that warrants regulatory attention. These omissions may lead to under-diagnosis or misattribution of alopecia to other causes, delaying appropriate intervention. From a regulatory perspective, this disconnect highlights the imperative for proactive post-marketing surveillance. Agencies like the FDA and EMA should leverage real-world data to issue safety communications and update prescribing information, particularly for high-risk, widely used drugs. Moreover, encouraging healthcare providers to report suspected DIA cases is crucial to improve signal detection and risk characterization.

In clinical practice, a thorough medication history is essential for any patient presenting with unexplained alopecia. Diagnosis of DIA requires establishing a temporal relationship between drug initiation and alopecia onset, along with the exclusion of other etiologies such as nutritional deficiencies, autoimmune diseases, or genetic predisposition. For confirmed cases of DIA, the first-line approach is discontinuing the causative drug, if clinically feasible, which often leads to hair regrowth within 3–6 months (Paus and Cotsarelis, 1999; Kinoshita et al., 2019). When drug cessation is insufficient or impractical, several therapies for AGA or AA can be considered for DIA, based on clinical experience and shared pathomechanisms. For instance, topical minoxidil, FDA-approved for AGA, promotes hair growth through vasodilation, antiandrogen effects, and modulation of the hair cycle phases (Gupta et al., 2022). Oral and sublingual minoxidil are also effective off-label options (Awad et al., 2023). Similarly, 5α-reductase inhibitors, a mainstay of AGA treatment, can be used, with combination therapy of finasteride and topical minoxidil demonstrating superior efficacy to monotherapy (Chen et al., 2020). Additionally, Janus kinase (JAK) inhibitors, including baricitinib, ritlecitinib, and deuruxolitinib, have proven effective for AA by prolonging the anagen phase and promote regrowth (Kim et al., 2025). Beyond these established options, emerging modalities like small-molecule inhibitors, biologics, and stem cell-based therapies also hold promise for more precise interventions (Kim et al., 2025).

This study represents the largest and most comprehensive pharmacovigilance analysis of DIA to date. Leveraging 2 decades of FAERS data, it identifies high-risk drugs, characterizes latency periods, and reveals demographic disparities. Our findings provide clinically actionable insights for risk mitigation and patient management. However, these results must be interpreted in the context of the inherent limitations of spontaneous reporting systems. First and foremost, disproportionality analysis can identify statistical associations indicative of potential safety signals, but not definitive cause-effect relationships. Second, FAERS data are subject to substantial reporting biases, including underreporting and demographic biases (such as the overrepresentation of reports from the United States and from females, as observed in our study) (Maciá-Martínez et al., 2016). Third, the lack of a definitive denominator makes it impossible to calculate true incidence rates or directly compare risks across drugs. Fourth, inconsistent data quality and frequent absence of key clinical details limited potential confounders adjustment. Finally, while our label review was confined to United States prescribing information, cross-regional comparisons may provide valuable insights into global disparities in safety communication. Despite these limitations, our study robustly identifies potential DIA risks. It provides a clear direction for more targeted and rigorous future research.

## 5 Conclusion

This comprehensive pharmacovigilance study systematically identified and characterized the risk of DIA across a wide range of medications. Our study identified 64 drugs with significant alopecia signals, predominantly from oncology, endocrine, and immunomodulatory classes. Notably, we observed distinct demographic patterns, with females and older adults being disproportionately affected, highlighting the importance of considering patient-specific factors in clinical practice. The study also revealed significant disparities in the latency period of DIA between males and females, underscoring the need for sex-specific monitoring protocols. Additionally, a substantial proportion (23.4%) of these high-risk drugs lacked documentation of alopecia in their official labels, indicating a concerning gap in current drug safety information. These findings enhanced the understanding of DIA epidemiology and provide practical guidance for improved patient risk assessment and management. Furthermore, they emphasized the necessity for regulatory agencies to incorporate real-world evidence into post-marketing surveillance and label updates. Overall, this study highlighted the importance of pharmacovigilance in identifying and mitigating the risk of DIA and supports the continuous improvement of drug safety profiles to enhance patient outcomes.

## Data Availability

Publicly available datasets were analyzed in this study. This data can be found here: https://fis.fda.gov/extensions/FPD-QDE-FAERS/FPD-QDE-FAERS.html.
