# Supplementary material for: Real-world pharmacovigilance insights into drug-induced risk of alopecia
Source: Front Pharmacol. 2025 Nov 28;16:1703423. doi: 10.3389/fphar.2025.1703423 (PMC12698529; doi:10.3389/fphar.2025.1703423)
Supplement: Supplementary file 1 [file Supplementaryfile1.docx]

Supplementary Material

# Supplementary Figures and Tables

## Supplementary Figures


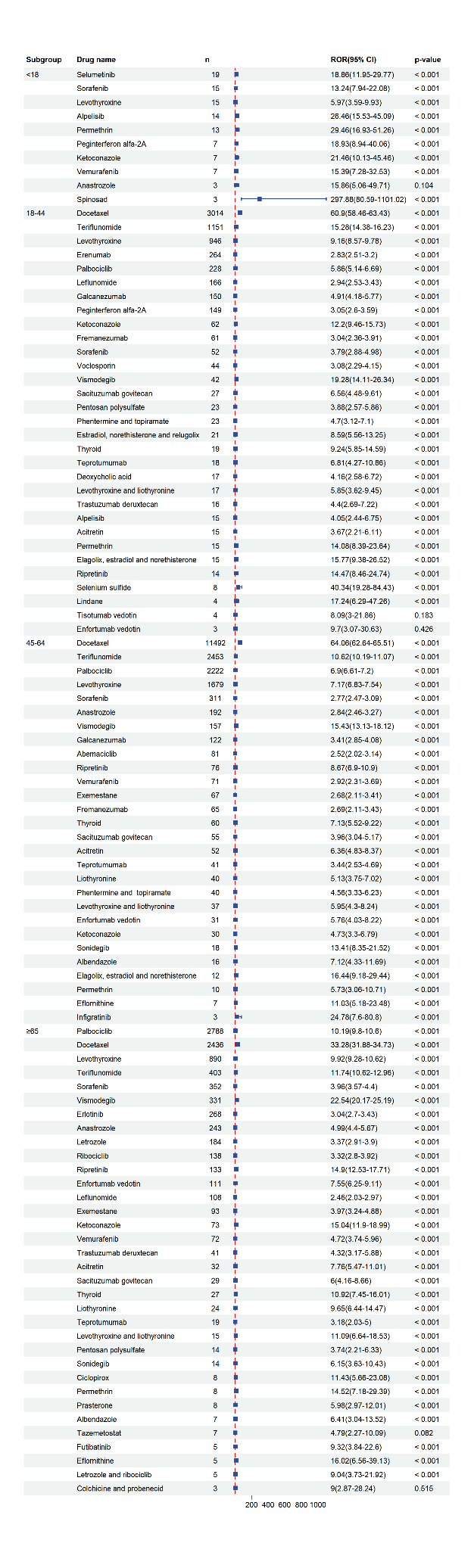


**Supplementary Figure S1. Forest plot of age subgroup analysis of drug-induced alopecia.**


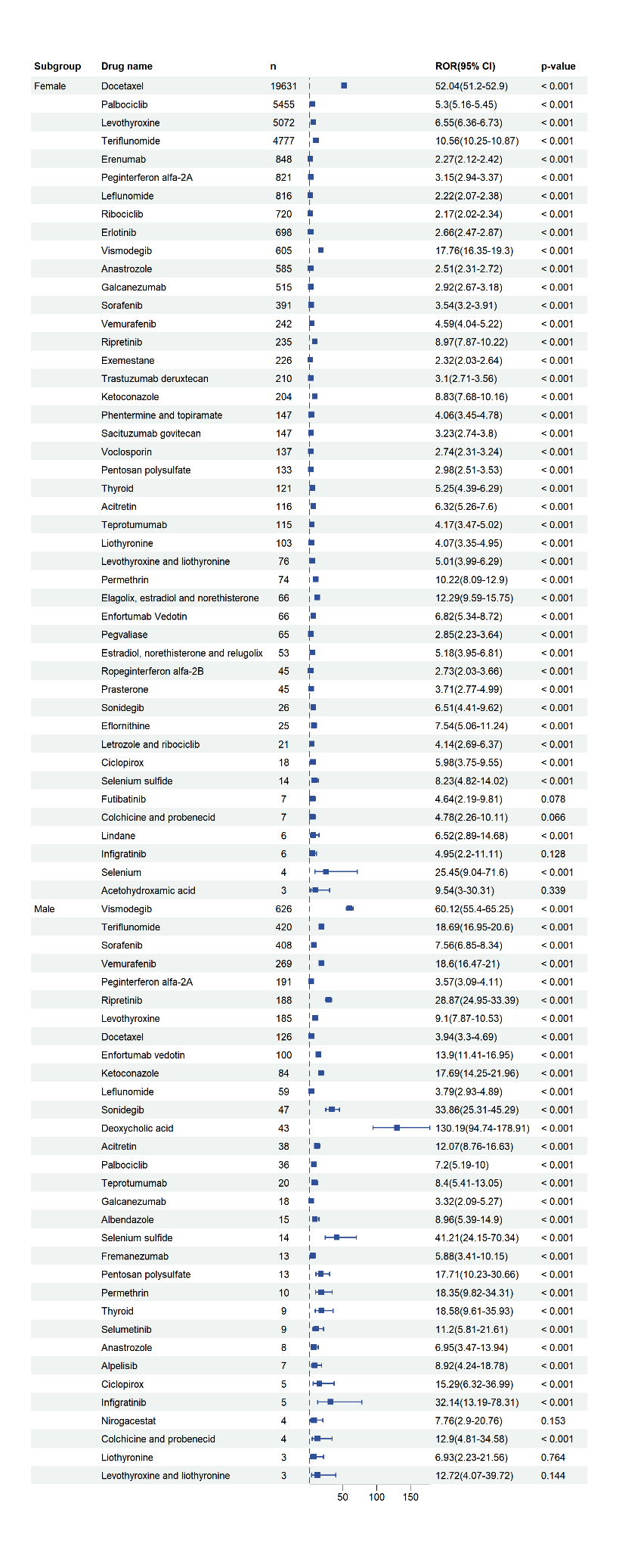


**Supplementary Figure S2. Forest plot of gender subgroup analysis of drug-induced alopecia.**

**
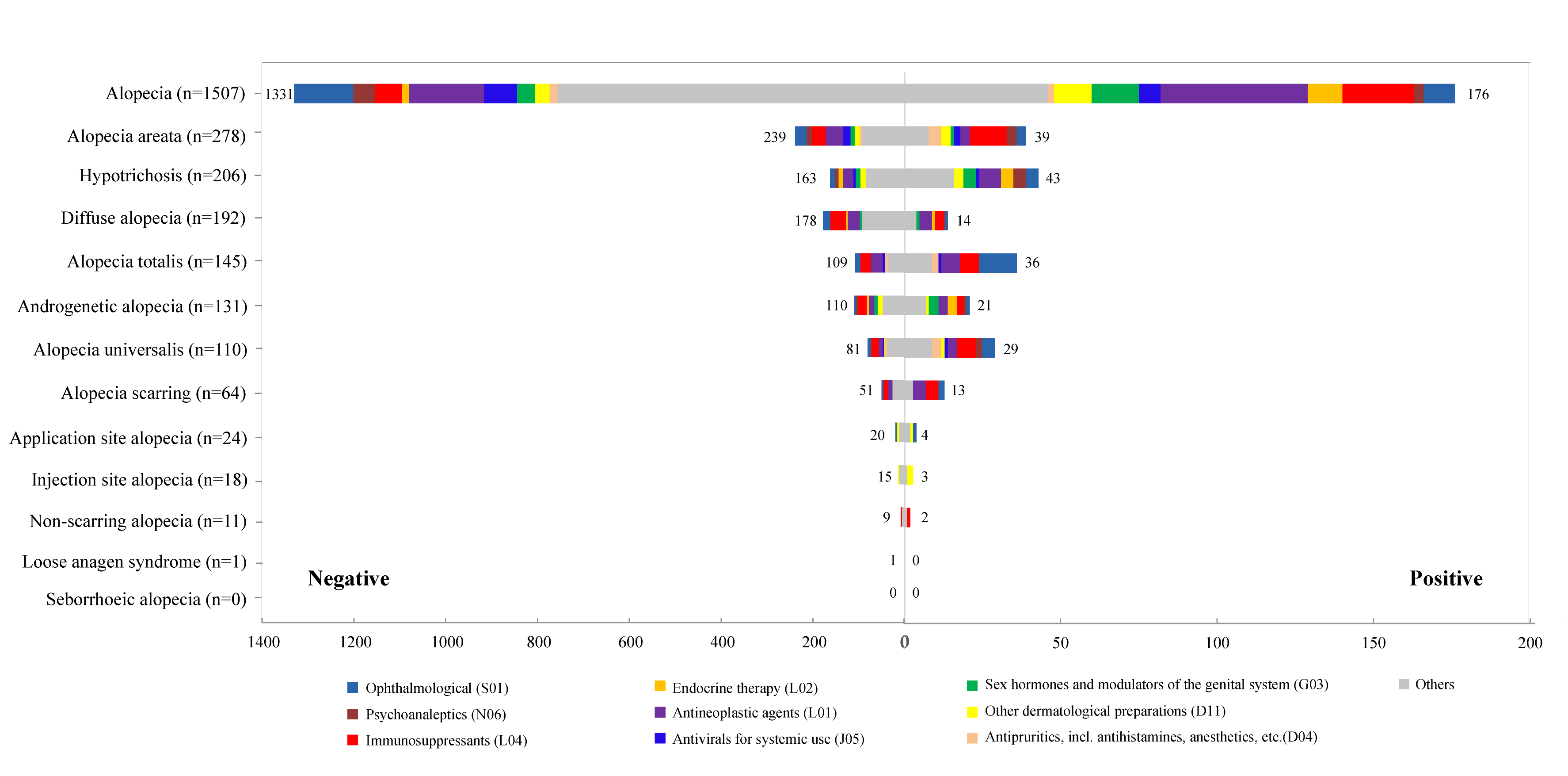
**

**Supplementary Figure S3. The positive-negative distribution of ADR signals for drugs at performed terms level and the corresponding distribution of drug class (anatomical therapeutic chemical classification system).**


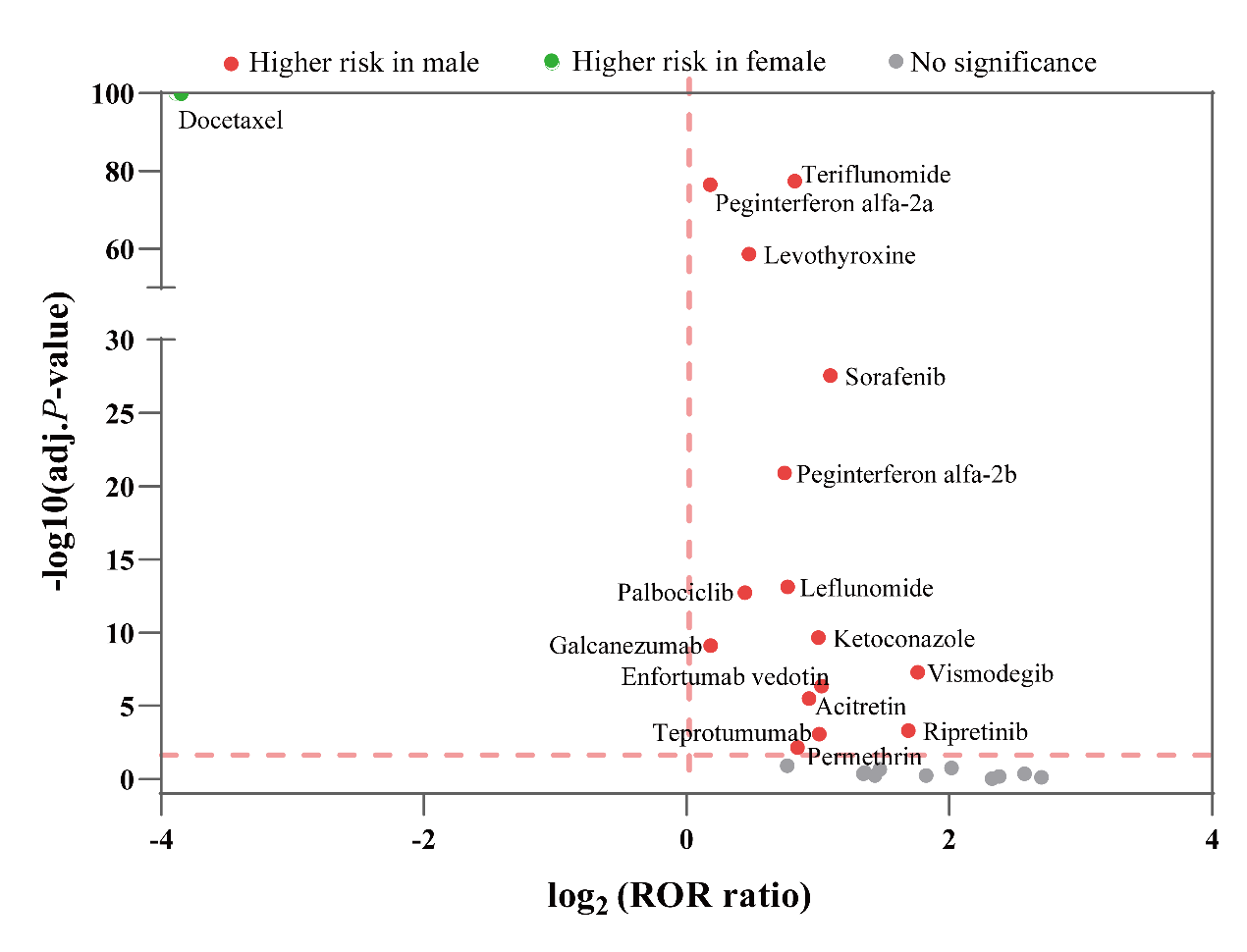


**Supplementary Figure S4.** **Volcano plot of gender distribution for drug-induced alopecia.**

## Supplementary Tables

**Supplementary Table S1 PTs contained in the narrow-scope search of “Alopecia (SMQ)”**

| **MedDRA Code** | **PTs** |
| --- | --- |
| 10001760 | Alopecia |
| 10001761 | Alopecia areata |
| 10001764 | Alopecia scarring |
| 10001766 | Alopecia totalis |
| 10001767 | Alopecia universalis |
| 10021126 | Hypotrichosis |
| 10059046 | Application site alopecia |
| 10068168 | Androgenetic alopecia |
| 10073736 | Diffuse alopecia |
| 10081519 | Injection site alopecia |
| 10082395 | Non-scarring alopecia |
| 10084141 | Seborrhoeic alopecia |
| 10085066 | Loose anagen syndrome |

Abbreviate: PT, preferred term; MedDRA, Medical Dictionary for Drug Regulatory Activities; SMQ, Standardized MedDRA Querie.

**Supplementary Table S2 Two-by-two contingency table for disproportionality analysis.**

| **Item** | **Target adverse events** | **Other adverse events** | **Total** |
| --- | --- | --- | --- |
| **Target drugs** | a | b | a + b |
| **Other drugs** | c | d | c + d |
| **Total** | a + c | b + d | a + b + c + d |

a, number of reports containing both the suspected drug and the suspected adverse drug reaction; b, number of reports containing the suspected adverse drug reaction with other medications (except the drug of interest); c, number of reports containing the suspected drug with other adverse drug reactions (except the event of interest); d, number of reports containing other medications and other adverse drug reactions.

**Supplementary Table S3** **Principles of disproportionality analysis and the criteria for signal detection**

| **Method** | **Calculation formula** | **﻿Criteria** |
| --- | --- | --- |
| **ROR** | $ROR=\frac{a / c}{b / d}$ | a ≥ 3  95%CI (lower limit) > 1 |
|  | $SE(lnROR)=\sqrt{\frac{1}{a}+\frac{1}{b}+\frac{1}{c}+\frac{1}{d}}$ |  |
|  | $95\%CI= e^{\ln\left( ROR \right)\pm1.96SE(lnROR)}$ |  |
| **PRR** | $PRR=\frac{a / (a+b)}{c / (c+d)}$ | a ≥ 3  PRR ≥ 2  $\chi2\geq4$ |
|  | $SE(lnPRR)=\sqrt{\frac{1}{a}-\frac{1}{a+b}+\frac{1}{c}-\frac{1}{c+d}}$ |  |
|  | $95\%CI= e^{\ln\left( PRR \right)\pm1.96SE(lnPRR)}$ |  |
|  | $\chi2 =\frac{{(ad-bc)}^{2}(a+b+c+d)}{( a+b)(a+c)(c+d)(b+d)}$ |  |
| **BCPNN** | $a_{exp} =\frac{\left( a+b \right)(a+c)}{( a+b+c+d)}$  $IC={log}_{2}\frac{a+0.5}{a_{exp}+0.5}$  $\mathrm{IC}_{025}=IC-3.3*\left( a+0.5 \right)^{-0.5}-2*\left( a+0.5 \right)^{-1.5}$ | IC_025_>0 |
| **MGPS** | $EBGM=\frac{a(a+b+c+d)}{\left( a+c \right)(a+b)}$ | EBGM_05_>2 |
|  | $\mathrm{EBGM}_{05}= e^{\ln\left( EBGM \right)}-1.64*{(\frac{1}{a}+\frac{1}{b}+\frac{1}{c}+\frac{1}{d})}^{-0.5}$ |  |

ROR, reporting odds ratio; PRR, proportional reporting ratio; BCPNN, Bayesian confidence propagation neural network; MGPS, Multi-item Gamma Poisson Shrinker; IC, information component; IC_025_, lower limit of the 95% CI of the IC; EBGM_05_, empirical Bayesian geometric mean lower 95% CI for the posterior distribution.

a, number of reports containing both the suspected drug and the suspected adverse drug reaction; b, number of reports containing the suspected adverse drug reaction with other medications (except the drug of interest); c, number of reports containing the suspected drug with other adverse drug reactions (except the event of interest); d, number of reports containing other medications and other adverse drug reactions.

**Supplementary Table S4. List of excluded drugs intended for alopecia treatment and their disproportionality analysis results.**

| **Drug name** | **Case reports** | **ROR(95% CI)** | **PRR(χ^2^)** | **IC(IC_025_)** | **EBGM**  **(EBGM_05_)** |
| --- | --- | --- | --- | --- | --- |
| Minoxidil | 7166 | 21.13 (20.62,21.65) | 19.87 (123729) | 4.26 (4.22) | 19.12 (18.66) |
| Methotrexate | 3579 | 2.16 (2.09,2.23) | 2.15 (2159.98) | 1.09 (1.04) | 2.13 (2.06) |
| Baricitinib | 137 | 3.31 (2.80,3.92) | 3.28 (218.30) | 1.72 (1.44) | 3.28 (2.77) |
| Clobetasol | 131 | 3.16 (2.66,3.75) | 3.14 (191.12) | 1.65 (1.37) | 3.13 (2.64) |
| Ethinylestradiol, iron and norethisterone | 70 | 2.64 (2.09,3.34) | 2.63 (70.74) | 1.39 (1.02) | 2.63 (2.08) |
| Ritlecitinib | 45 | 10.30 (7.65,13.86) | 9.99 (365.37) | 3.32 (2.63) | 9.99 (7.42) |
| Drospirenone and estradiol | 17 | 6.41 (3.96,10.36) | 6.30 (75.98) | 2.65 (1.59) | 6.30 (3.89) |

Note: The above seven drugs, which are indicated for the treatment of alopecia (e.g., alopecia areata, androgenetic alopecia), were excluded from the main analysis. Their positive signals are considered more likely to represent reports of inadequate therapeutic efficacy rather than true adverse drug reactions.

Abbreviations: ROR, reporting odds ratio; PRR, proportional reporting ratio; IC, information component; IC_025_, lower limit of the 95% CI of the IC; EBGM_05_, empirical Bayesian geometric mean lower 95% CI for the posterior distribution.

**Supplementary Table S5. Time to onset for drug-induced alopecia by different drugs based on gender stratification.**

| Drug name | Female  Median (IQR) | Male  Median (IQR) | *P* value |
| --- | --- | --- | --- |
| Acitretin | 14.00 (0.00 - 92.00) | 63.00 (0.00 - 175.00) | 0.46 |
| Albendazole | 6.00 (1.50 - 21.50) | 14.00 (5.00 - 16.00) | 0.96 |
| Alpelisib | 10.00 (0.00 - 76.00) | 0.00 (0.00 - 32.00) | 0.17 |
| Deoxycholic acid | 1.00 (1.00 - 1.00) | 21.00 (10.00 - 38.00) | 0.48 |
| Docetaxel | 114.00 (27.00 - 282.00) | 8.00 (3.00 - 25.00) | 0.00 |
| Enfortumab vedotin | 15.00 (7.00 - 28.00) | 8.50 (6.00 - 16.00) | 0.06 |
| Erenumab | 41.50 (5.00 - 122.50) | 73.00 (38.50 - 208.00) | 0.30 |
| Erlotinib | 49.00 (9.00 - 143.00) | 18.00 (4.50 - 121.50) | 0.39 |
| Fremanezumab | 47.00 (4.00 - 143.00) | 35.00 (0.00 - 187.00) | 0.54 |
| Fumaric acid | 27.00 (0.00 - 136.00) | 30.00 (1.00 - 139.00) | 0.94 |
| Galcanezumab | 35.00 (7.00 - 98.50) | 96.50 (37.00 - 203.50) | 0.43 |
| Ketoconazole | 0.00 (0.00 - 1.50) | 0.00 (0.00 - 0.00) | 0.45 |
| Leflunomide | 54.00 (4.00 - 119.00) | 108.00 (79.00 - 245.00) | 0.13 |
| Levothyroxine | 28.00 (3.00 - 100.00) | 30.00 (4.00 - 609.00) | 0.67 |
| Levothyroxine and liothyronine | 57.50 (8.00 - 120.00) | 37.00 (0.00 - 74.00) | 0.44 |
| Liothyronine | 4.00 (0.00 - 58.00) | 83.00 (83.00 - 83.00) | 0.44 |
| Nirogacestat | 6.50 (0.00 - 14.00) | 0.00 (0.00 - 2.00) | 0.18 |
| Palbociclib | 75.50 (13.00 - 293.00) | 114.00 (10.00 - 153.00) | 0.98 |
| Peginterferon alfa-2A | 51.00 (7.00 - 118.00) | 32.50 (0.00 - 148.00) | 0.80 |
| Pegvaliase | 358.50 (187.00 - 530.00) | 176.00 (176.00 - 176.00) | 0.54 |
| Permethrin | 0.00 (0.00 - 2.00) | 0.00 (0.00 - 0.00) | 0.10 |
| Prasterone | 0.00 (0.00 - 7.00) | 92.00 (92.00 - 92.00) | 0.53 |
| Ribociclib | 17.50 (3.00 - 131.00) | 46.00 (13.00 - 77.00) | 0.63 |
| Ripretinib | 44.00 (4.00 - 167.00) | 26.50 (5.00 - 102.00) | 0.53 |
| Ropeginterferon alfa-2B | 18.50 (0.00 - 75.00) | 15.00 (12.00 - 110.00) | 0.64 |
| Selenium sulfide | 28.00 (0.00 - 124.00) | 0.00 (0.00 - 0.00) | 0.20 |
| Selumetinib | 21.00 (10.00 - 137.00) | 45.00 (42.00 - 129.00) | 0.68 |
| Sonidegib | 21.00 (0.00 - 32.00) | 84.50 (17.00 - 222.00) | 0.37 |
| Sorafenib | 7.00 (0.00 - 16.00) | 7.00 (2.00 - 20.00) | 0.46 |
| Tazemetostat | 113.00 (0.00 - 226.00) | 0.00 (0.00 - 0.00) | 1.00 |
| Teprotumumab | 68.00 (0.00 - 149.00) | 167.00 (77.00 - 181.00) | 0.17 |
| Teriflunomide | 69.50 (15.50 - 203.00) | 97.00 (21.00 - 285.00) | 0.17 |
| Thyroid | 156.50 (9.00 - 1188.00) | 89.00 (0.00 - 217.00) | 0.25 |
| Trastuzumab deruxtecan | 14.00 (0.00 - 178.00) | 43.00 (43.00 - 43.00) | 0.65 |
| Vemurafenib | 10.50 (3.00 - 28.00) | 9.50 (4.50 - 17.50) | 0.80 |
| Vismodegib | 48.50 (16.00 - 139.00) | 77.00 (14.00 - 159.00) | 0.30 |
| Voclosporin | 30.00 (2.00 - 87.00) | 0.00 (0.00 - 0.00) | 0.18 |
